# Assembly and Interrogation of Alzheimer’s Disease Genetic Networks Reveal Novel Regulators of Progression

**DOI:** 10.1371/journal.pone.0120352

**Published:** 2015-03-17

**Authors:** Soline Aubry, William Shin, John F. Crary, Roger Lefort, Yasir H. Qureshi, Celine Lefebvre, Andrea Califano, Michael L. Shelanski

**Affiliations:** 1 Taub Institute for Research on Alzheimer's Disease & the Aging Brain and the Department of Pathology & Cell Biology, Columbia University, New York, NY, 10032, United States of America; 2 Department of Systems Biology, Columbia University, New York, NY, 10032, United States of America; 3 Herbert Irving Comprehensive Cancer Center, Columbia University, New York, NY, 10032, United States of America; 4 Department of Biological Sciences, Columbia University, New York, NY, 10027, United States of America; 5 Inserm Unit U981, Institut Gustave Roussy, 94805, Villejuif, France; National Center for Geriatrics and Gerontology, JAPAN

## Abstract

Alzheimer’s disease (AD) is a complex multifactorial disorder with poorly characterized pathogenesis. Our understanding of this disease would thus benefit from an approach that addresses this complexity by elucidating the regulatory networks that are dysregulated in the neural compartment of AD patients, across distinct brain regions. Here, we use a Systems Biology (SB) approach, which has been highly successful in the dissection of cancer related phenotypes, to reverse engineer the transcriptional regulation layer of human neuronal cells and interrogate it to infer candidate Master Regulators (MRs) responsible for disease progression. Analysis of gene expression profiles from laser-captured neurons from AD and controls subjects, using the Algorithm for the Reconstruction of Accurate Cellular Networks (ARACNe), yielded an interactome consisting of 488,353 transcription-factor/target interactions. Interrogation of this interactome, using the Master Regulator INference algorithm (MARINa), identified an unbiased set of candidate MRs causally responsible for regulating the transcriptional signature of AD progression. Experimental assays in autopsy-derived human brain tissue showed that three of the top candidate MRs (YY1, p300 and ZMYM3) are indeed biochemically and histopathologically dysregulated in AD brains compared to controls. Our results additionally implicate p53 and loss of acetylation homeostasis in the neurodegenerative process. This study suggests that an integrative, SB approach can be applied to AD and other neurodegenerative diseases, and provide significant novel insight on the disease progression.

## Introduction

Alzheimer’s disease (AD) is the most common form of dementia in the elderly and is projected to affect over 100 million people worldwide by 2050 [1]. At the pathological level, it is characterized by neuronal loss, extracellular plaques of Aβ and intracellular neurofibrillary tangles composed of hyper-phosphorylated tau [2]. Currently there are no effective approaches to prevent, cure or even slowdown the progression of the disease. Classical genetic and biochemical approaches have implicated specific molecular pathways, such as those involved in APP processing/ Aβ generation [3–5], dendritic spine alterations [6,7], and inflammation [8]. More recently, multiple high-throughput technologies (transcriptomics, genomics, proteomics) have been applied and have led to the discovery of new processes that are dysregulated in AD, such as those involved in protein misfolding, altered lipid and cholesterol homeostasis or oxidative stress [9–12]. Yet, a comprehensive understanding of AD pathogenesis remains elusive and will likely require an integrative, regulatory network based analysis [13] of these distinct pathophysiological processes to understand their inter-connectivity and their role in disease progression.

Systems Biology (SB) predicates the use of regulatory-model based methodologies, as opposed to purely statistical association approaches, to support the integrative, unbiased interrogation of large datasets to study their global behavior rather than studying the effect of individual genes. An important limitation of classical approaches, relying on statistical association rather than causal regulatory model analysis, is the inability to distinguish between primary causal disease drivers and secondary (passenger) non-causal events. Indeed, as previously shown for the elucidation of human malignancy drivers, these approaches are very effective at identifying both individual and synergistic Master Regulators (MRs) genes that are both necessary and/or sufficient to induce presentation of a specific pathophysiological phenotype, while discarding the majority of passenger genes. These methods, for instance, have led to elucidation of experimentally validated drivers of human disease and associated mechanisms, including the synergistic pair C/EBP and STAT3 in the mesenchymal subtype of glioma [14], the triplet TLX1, TLX3, and RUNX1 in acute lymphoblastic leukemia tumorigenesis [15], the pair FOXM1 and MYB in formation of germinal centers [16], and the AKT1 protein in driving glucocorticoid resistance in T cell acute lymphoblastic leukemia [17], among many others, see [13] for a review.

In sharp contrast, model-based, SB approaches are just beginning to be applied to diseases of the nervous system. In the context of AD, one study using “weighted gene co-expression network analysis” found AD-related coexpression modules involved in immune response and in synaptic and metabolic functions [18]. More recently, expression profiles from whole-brain samples were used to construct a gene-regulatory network that implicated immune- and microglia-associated genes in the progression of AD [19]. However, the genetic determinants and functional effectors of AD that are specific to the neural compartment of the brain, which is ultimately the one affected by the disease, remain elusive and have yet to be studied using a network biology approach. Here, we propose that regulatory network methods that have produced significant discoveries in human neoplasia can be effectively applied to neurobiology and may provide an important discovery tool for elucidating drivers and effectors of neurodegenerative processes. In particular, we reasoned that interactome-based approaches using neuron-specific regulatory networks could help unveil novel genes contributing to AD pathogenesis in this cellular context.

Since an accurate, neuron-specific model of transcriptional regulation for AD is still elusive, we used the Algorithm for the Reconstruction of Accurate Cellular Networks (ARACNe) to analyze a large-scale gene expression profile dataset obtained from neurons isolated by laser-capture microdissection from human AD and control subjects [Gene Expression Omnibus dataset, GSE5281 and GSE9770 [20,21]]. This analysis yielded a rich regulatory network representing the transcriptional layer of the human neuronal *interactome*—i.e., the genome-wide repertoire of transcription-factor/target interactions that comprise the regulatory logic of neuronal cells. This regulatory network model was then interrogated by the Master Regulator INference algorithm (MARINa) using gene expression signatures representing distinct brain regions and AD stages to infer candidate MRs of clinically relevant phenotypes. Biochemical and neuropathological validation studies suggest a role in AD pathogenesis for three novel MRs inferred among the most significant ones by MARINa (YY1, p300 and ZMYM3), both at early and late stages of disease progression. Taken together, these findings highlight novel specific pathological processes potentially leading to neuronal dysfunction and toxicity, such as an increased stabilization of p53 and loss of acetylation homeostasis. Furthermore, they suggest that, similar to what has happened in the study of human malignancies, unbiased analysis of genome-wide regulatory networks can further our understanding of pathogenesis in AD and other neurodegenerative disorders.

## Results

### Selection and assessment of the gene expression profiles dataset

**Fig 1:**
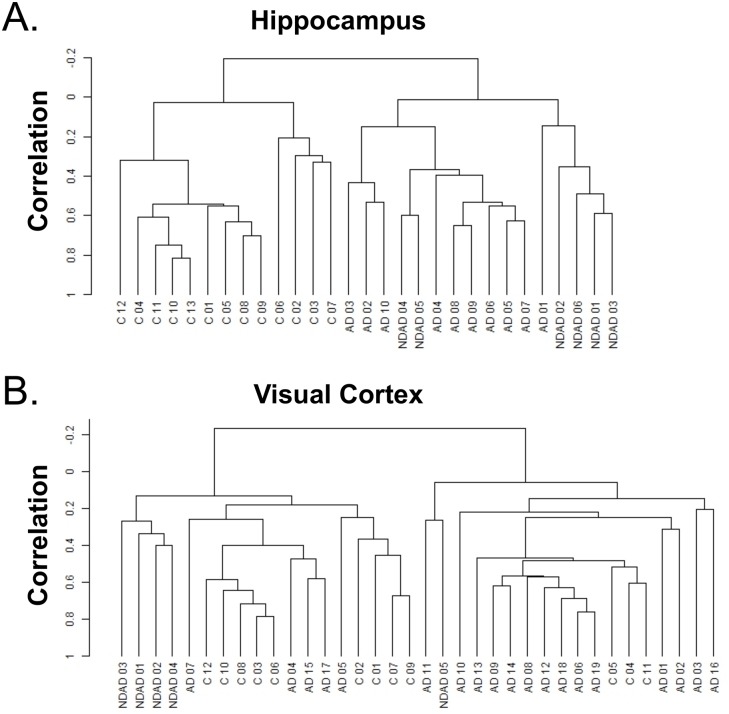
Cluster dendrograms for hippocampus and visual cortex samples. Bioinformatic analysis reveals a high degree of clustering of Alzheimer disease (AD), non-demented AD (NDAD) and control (C) in the hippocampus but not the primary visual cortex. Dendrogram showing clustering of (*A*) the hippocampus and (*B*) primary visual cortex samples using centered correlation and average linkage.

To identify neuron-specific Master Regulators (MRs) as drivers of Alzheimer’s disease (AD) initiation and progression, gene expression signatures were generated from laser-captured neurons from AD and control cases, encompassing both pathological and clinical phenotypes. Specifically, we selected the Liang et al. dataset, which comprises gene expression profiles from laser-capture microdissected (LCM) cortical neurons isolated from six anatomically and functionally distinct postmortem human brain regions representing 14 controls, 10 non-demented individuals with AD-type changes in their brains at autopsy (NDAD), and 34 demented individuals with the histopathological confirmation of AD [20,21] (S1 Table). To assemble the regulatory model (interactome), we used the full set of 193 gene expression profiles representing the entorhinal cortex (EC), hippocampus (HIP), middle temporal gyrus (MTG), posterior cingulate (PC), superior frontal gyrus (SFG) and visual cortex (VCX) of these individuals. Cluster analysis revealed that samples from regions known to be severely affected in AD (e.g., HIP, EC or MTG) show tight clustering according to diagnosis whereas samples from regions relatively less affected (i.e., VCX) do not cosegregate (Fig. 1 and S1 Fig.). Therefore, unbiased clustering analysis confirms the reproducibility of clinically relevant molecular phenotypes and suggests that patient stratification into these three diagnostic categories is biologically relevant, as supported by common molecular features. Thus, the categories “Control”, “NDAD” and “AD” were used to inform follow up analyses.

### Construction of the human neuronal transcriptional interactome

The Algorithm for the Reconstruction of Accurate Cellular Networks (ARACNe) [22] was previously developed to reconstruct the transcriptional regulatory logic of specific cellular contexts. This logic comprises the set of regulatory targets, or *regulon*, of each transcription factor (TF) or other transcriptional regulator of gene expression. For simplicity, we will use the term TF to indicate both classes. Analysis of these 193 gene expression profiles by ARACNe yielded the first human causal transcriptional network for cortical neurons, representing 488,353 individual transcriptional interactions between 3,758 TFs expressed in this context and their transcriptional targets.

### Identification of candidate Master Regulators using MARINa

We then used the previously developed Master Regulator INference algorithm (MARINa) to interrogate regulatory networks to identify candidate MR genes [14,16]. These represent regulators that are sufficient and/or necessary causal determinants of a specific phenotypic difference. MR candidates are identified by MARINa based on the enrichment of their ARACNe-predicted targets in the differentially expressed genes of a specific phenotype, similar to using a gene reporter assay approach to detect aberrant activity of a TF. The difference is that instead of using a single reporter controlled by the promoter of a TF-target gene, we use the expression of all the ARACNe-inferred targets of the TF. This allows identification of regulators whose aberrant activity is post-translationally determined, which could not be identified by differential expression, thus overcoming the limit of traditional analysis.

**Fig 2:**
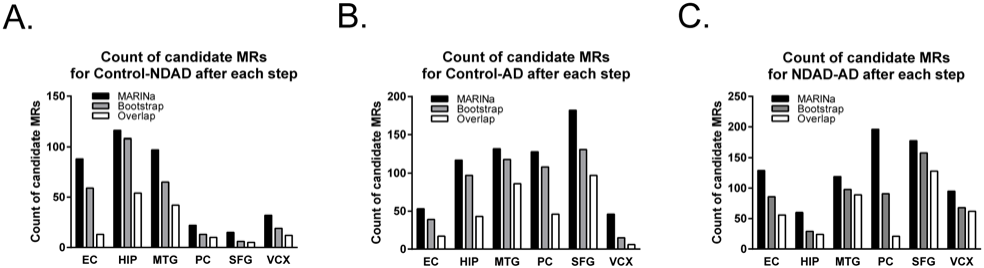
Count of candidate MRs per region and per comparison groups after each computational analysis. Master Regulator Inference analysis (MARINa) reveals different numbers of candidate Master Regulators (MRs) by brain region and comparison group. Count of candidate MRs in (*A*) control v. Alzheimer’s disease (AD), (*B*) control v. non-demented Alzheimer’s disease (NDAD) and (*C*) non-demented Alzheimer’s disease (NDAD) v. Alzheimer’s disease (AD) after each analysis. EC = entorhinal cortex, HIP = hippocampus, MTG = middle temporal gyrus, PC = posterior cingulate, SFG = superior frontal gyrus, VCX = visual cortex.

MARINa analysis was performed on 18 distinct gene expression profile signatures, representing three distinct phenotypic differences (Control→NDAD, NDAD→AD and Control→AD) across each of the six brain regions. Candidate MRs were first selected based on their statistical significance and then sorted by their Differentially Expressed Targets Odds Ratio (DETOR) [16]. The latter represents the density of targets in the leading edge of the GSEA analysis compared to the remaining range of expression and is a direct measure of the regulatory impact of the MR. These analyses result in relatively small ranked-lists containing TFs that are most likely to be responsible for the phenotypic difference in the brain region of interest. We also reasoned that candidate MRs for a region specific signature should be independent from the number and specific selection of the samples in the analysis. Thus, to filter out false positive MRs, we used bootstrapping on the 18 representative gene expression profile signatures, each time randomly sampling only 70% of the total samples representing the signature, with replacement. Candidate MRs that failed to be identified in all 10 bootstrap runs were then considered false-positives and were excluded from further consideration. The results of the bootstrapping step on the count of candidate MRs are summarized in Fig. 2. In the control v. NDAD phenotypic change, high numbers of candidates are found in EC, HIP and MTG, which are regions showing early pathology in AD and are also affected in aging to variable degrees. In contrast, in the control v. AD phenotypic change, we observe a positive correlation between the count of candidate MRs and the regional progression of AD. Finally, comparing NDAD and AD allows for the highlighting of MRs mostly responsible for dementia progression, as pathological hallmarks are already present in NDAD patients. Interestingly, the highest counts are found in EC, MTG, PC and SFG and a very low count is found in HIP, showing that in this region a small number of MRs are predicted to drive the phenotypic differences between NDAD and AD patients. This is a common finding for this type of analysis, such that signatures representing phenotype-relevant events produce a greater number of highly statistically significant MRs than signatures that are not the result of specific regulatory events. To this extent, PC and SFG are the two regions that differ significantly between the two phenotypic changes of interest (control v. AD and control v. NDAD). As a negative control, VCX was found to show a significant decrease in the number of candidate MRs in all three phenotypic changes, thus confirming its less representative role in AD.

### Selection of candidate MRs for biochemical validation

In order to prioritize MRs for further biochemical validation, we made two assumptions. First, we reasoned that AD should be reflected across a multitude of genetic programs that are either brain region specific or common to multiple regions. However, we anticipated that focusing on region-specific programs would lead to discovery of many idiosyncratic false-positive MRs (e.g., those regulating region-specific downstream programs from the true MRs or programs activated within specific regions in response to neurodegeneration), whereas focusing on MRs conserved across multiple regions should be more specific to bona fide upstream AD drivers. Hence, for each phenotypic difference, we selected candidate MRs conserved across more than one region, thus eliminating candidate MRs identified only in one region. Fig. 2 summarizes this analysis and S2 through S7 Tables display the final lists of MRs on a region and transition-specific basis, after this step.

Second, we assumed that candidate MRs regulating hippocampus-specific signatures would be of particular relevance, as this region plays a central role in memory and is one of the first ones affected in AD [25–27]. Two strategies were thus followed in parallel: either prioritizing candidates that are most highly ranked in HIP (strategy 1), or prioritizing those that show up in the highest number of regions, including HIP (strategy 2). Through these assumptions, we reduced our original MR set to about 20 candidates per strategy (Tables 1 and 2). Two of the highest ranked genes from each strategy, for which appropriate reagents were available, were then selected for further validation in human brain tissue, by immunohistochemistry and Western blot analyses. These include the E1A binding protein p300 (*EP300*), Ying Yang 1 (*YY1*), the Zinc Finger, MYM-Type 3, (*ZMYM3*), and the Myocyte Enhancer Factor 2D (*MEF2D*).

**Table 1:** List of candidate MRs for each comparison (Control v. NDAD and Control v. AD) in strategy 1 (highly ranked in HIP).

Gene Name	Probe Number	NES	Odds Ratio
*Alzheimer’s disease*			
EP300	202221 s at	1.98	151.63
YY1	201901 s at	1.97	133.82
BUD31	205690 s at	-1.88	94.58
ILF3	208930 s at	2.09	65.61
TRIM27	212118 at	-1.88	56.33
ZDHHC21	233216 at	1.99	55.42
ZFC3H1	213065 at	-1.96	47.31
ZNF75A	227670 at	-1.89	37.53
ZNF410	202010 s at	-1.89	33.94
ZNF451	215012 at	1.89	27.98
*Non-demented Alzheimer’s disease*			
KDM5A	215698 at	1.98	191.76
TFAM	203176 s at	2.12	183.43
MEF2A	208328 s at	2.00	104.84
ZNF800	227101 at	2.00	102.54
ZFAND5	217741 s at	2.00	75.42
THRA	204100 at	-1.88	72.81
ZCCHC2	219062 s at	2.05	40.86
EP300	202221 s at	1.90	38.00
NFIA	224976 at	-1.90	36.78
BCL11A	219498 s at	1.99	36.54
BCL11A	222891 s at	1.97	30.92
SALL2	213283 s at	-1.97	25.00
ZBTB16	205883 at	-1.96	23.91
THRA	35846 at	-1.99	21.93

*MR* master regulator, *NES* normalized enrichment score, *HIP* hippocampus, *AD* Alzheimer’s disease, *NDAD* non-demented Alzheimer disease

**Table 2:** List of candidate MRs for each comparison (Control v. NDAD and Control v. AD) in strategy 2 (enriched in HIP and at least 2 other regions).

Gene Name	Probe Number	# regions	NES(HIP)	Odds Ratio(HIP)
*Alzheimer’s disease*				
THRA	1316_at	4	1.97	105.009
BBX	223134_at	4	1.97	55.002
NFAT5	208003_s_at	4	1.98	138.656
EP300	202221_s_at	3	1.98	151.633
ZFR	201856_s_at	3	1.99	141.377
ZNF710	39891_at	3	1.99	134.643
ZNF609	212620_at	3	1.98	118.809
ZNF785	1554770_x_at	3	1.89	117.578
ZNF562	219163_at	3	1.91	109.206
KHSRP	204372_s_at	3	1.94	102.221
BUD31	205690_s_at	3	-1.88	94.581
PHF3	215718_s_at	3	1.99	82.351
ZNF264	205917_at	3	1.90	60.619
TRIM27	212118_at	3	-1.88	56.330
ZDHHC21	233216_at	3	1.99	55.417
LMO3	204424_s_at	3	-1.94	34.285
ZCCHC17	223107_s_at	3	-1.83	31.673
*Non-demented Alzheimer’s disease*				
ZMYM3	1554171 at	6	1.97	45.92
MEF2D	225641 at	5	2.00	74.56
RBPJ	211974 x at	5	-2.01	20.13
THRA	35846 at	4	-1.99	21.93
ZCCHC2	219062 s at	4	2.05	40.86
ZNF780B	244818_at	3	1.88	188.16
ZDHHC21	233216_at	3	1.95	40.79
NFIA	224976_at	3	-1.90	36.78
BCL11A	219498_s_at	3	1.99	36.54
ZNF320	229614_at	3	1.94	33.66
CTNP1	213980_s_at	3	-2.29	8.96

*MR* master regulator, *NES* normalized enrichment score, *HIP* hippocampus, *AD* Alzheimer’s disease, *NDAD* non-demented Alzheimer disease

Finally, we asked whether the expression levels of these selected candidates in the laser-captured neuron dataset correlated with that observed in mRNA obtained from whole human brain tissue (S3 Fig.). For *YY1*, *EP300* and *ZMYM3*, this analysis revealed an increase of mRNA levels in AD cases compared to controls, suggesting that these two systems are comparable for these genes and therefore justifying utilization of whole tissue for further analyses. MEF2D shows no difference in the expression levels in the LCM dataset and the whole brain extracts, however, this finding is compatible with our analysis, which focuses on inferred activity rather than differences in expression levels. When investigated further using immunoblot, immunohistochemistry and binding assays, the experiments did not show consistent differences for both MEF2D and phospho-MEF2D (unpublished data). Thus, only the results for *YY1*, *EP300* and *ZMYM3* are presented below.

### Experimental validation of candidate MRs in AD brain

#### 1. YY1

**Fig 3:**
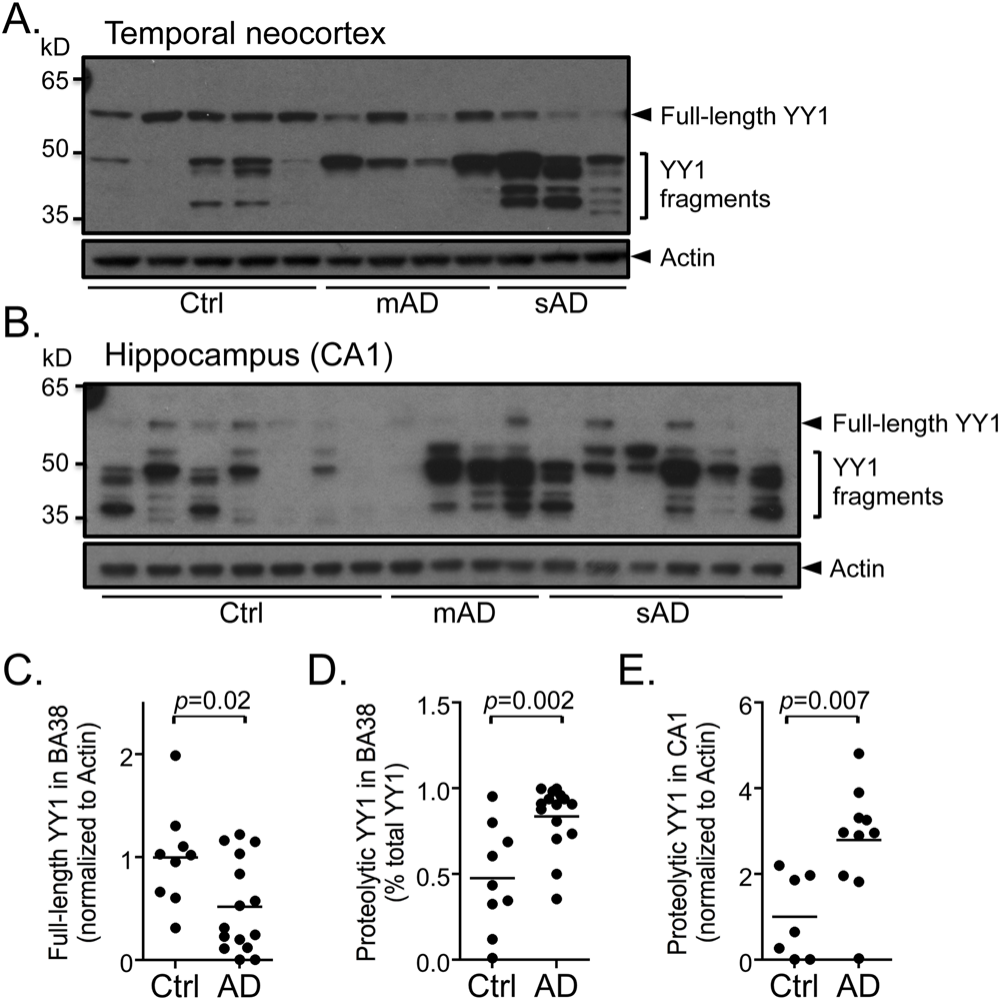
Increased proteolysis of YY1 is observed in post-mortem Alzheimer’s disease (AD) brain tissue. (*A*, *B*) Representative immunoblots using nuclear fractions from 12 human brain tissue samples from temporal neocortex (BA38) and 17 from hippocampus (CA1) show nuclear full-length and proteolytic fragments of YY1 in control (Ctrl), moderate Alzheimer’s disease (mAD) and severe Alzheimer’s disease (sAD) cases. Densitometric analysis of immunoblots from BA38 shows (*C*) a significant decrease (*p* = 0.02) in the full-length YY1 protein (60 kDa) in AD (*n* = 15, mAD + sAD) and (*D*) a significant increase (*p* = 0.002) in the proportion of proteolytic fragments (35–50 kDa) compared to controls (*n* = 9). Densitometric analysis of immunoblots from CA1 also reveals a significant increase (*p* = 0.007) in YY1 proteolytic fragments in AD (*n* = 10, mAD + sAD) compared to controls (*n* = 7). Comparisons were made using a Student’s t-test.

**Fig 4:**
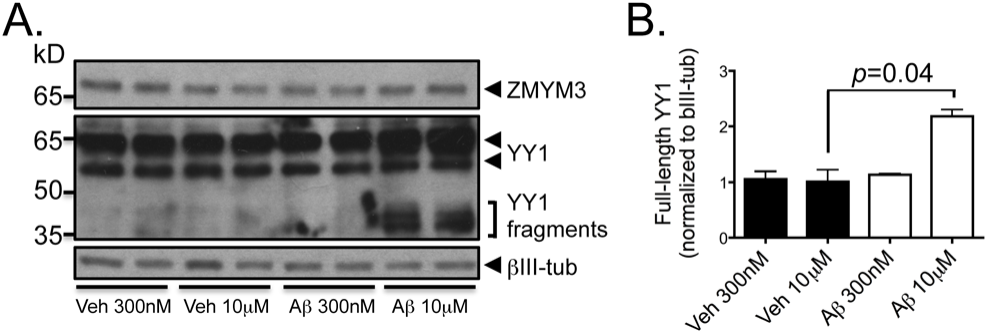
Exogenous treatment of rat primary hippocampal cultures with cytotoxic concentrations of dodecomeric preparations of Aβ (Aβ_dod_) results in increased YY1 protein levels and proteolysis. (*A*) Western blot analyses of extracts from cells treated with Aβ_dod_ for 6 hr show an increase in full-length YY1 as well as the appearance of proteolytic cleavage fragments between 35 and 50 kDa at 10 μM but not 300 nM. (*B*) Densitometric analysis of the full-length 65 kDa YY1 band shows a statistically significant increase in full-length YY1 in cells treated with 10 μM Aβ_dod_ compared to control (*p* = 0.04). Comparisons were made using a Student’s t-test. Veh = vehicle, tub = tubulin.

YY1 has been implicated in a variety of cellular processes, including proliferation, differentiation, development and apoptosis [28]. Immunohistochemical staining for YY1 shows no significant differences between AD and controls (S4A and S4B Fig.). To determine whether there are differences in the expression of YY1 protein in AD brain tissue, the levels of the protein were measured by quantitative immunobloting in samples from two different regions to account for regional variability, the hippocampus and the temporal neocortex, and from two different stages of AD, labeled “moderate” (mAD) and “severe” AD (sAD), to study potential changes associated with disease severity. Moderate cases differ from NDAD at the clinical level. Analysis of nuclear extracts from the temporal neocortex (BA38) and hippocampus (CA1) from autopsy brain tissue reveals multiple bands between 35 and 50 kD as well as a consistent 60 kDa band corresponding to full-length YY1 (Fig. 3A and 3B). Since YY1 is a caspase substrate and caspases are active in AD [29–32], we reasoned that the low molecular weight bands might be cleavage products. Indeed, recombinant caspases 3 and 7 hydrolyze endogeneous YY1 into fragments of 35 kDa comparable to those observed in human brains (S5 A and S5B Fig.). Furthermore, immunoblots prepared using lysates from primary rat hippocampal neuronal cultures treated with apoptotic concentration of Aβ dodecamers (Aβ_dod_) that activate caspases 3/7 and calpain proteases [33] leads to the appearance of two bands between 35 and 50 kDa that are not observed at subapoptotic concentrations (Fig. 4A and 4B). The observed increase in the full-length YY1 protein might also be indicative of a change in reactivity. Peptide competition blocks the appearance of these low molecular weight bands, further supporting antibody specificity (S5C Fig.). Analysis of 24 nuclear extracts from temporal neocortex (BA38) shows a significant decrease of the full-length YY1 in AD (*p* = 0.02) and a significant increase of the proteolytic fragments as compared to controls (*p* = 0.002) (Fig. 3C and 3D). No significant differences are observed between mAD and sAD cases. The CA1, which is more severely affected than the temporal neocortex, shows a stronger reduction of YY1 full-length protein (Fig. 3B). The proteolytic fragments are also significantly increased in AD cases compared to controls (*p* = 0.007) (Fig. 3E). Together, these results suggest that YY1 is cleaved in early stages of AD and that proteolytic fragments are predominant when the pathology is severe. Additionally, apoptotic concentrations of Aβ regulate YY1 and promote its cleavage into fragments of similar molecular weight as those observed in human brain tissue suggesting that YY1 is downstream to Aβ activity.

#### 2. p300

**Fig 5:**
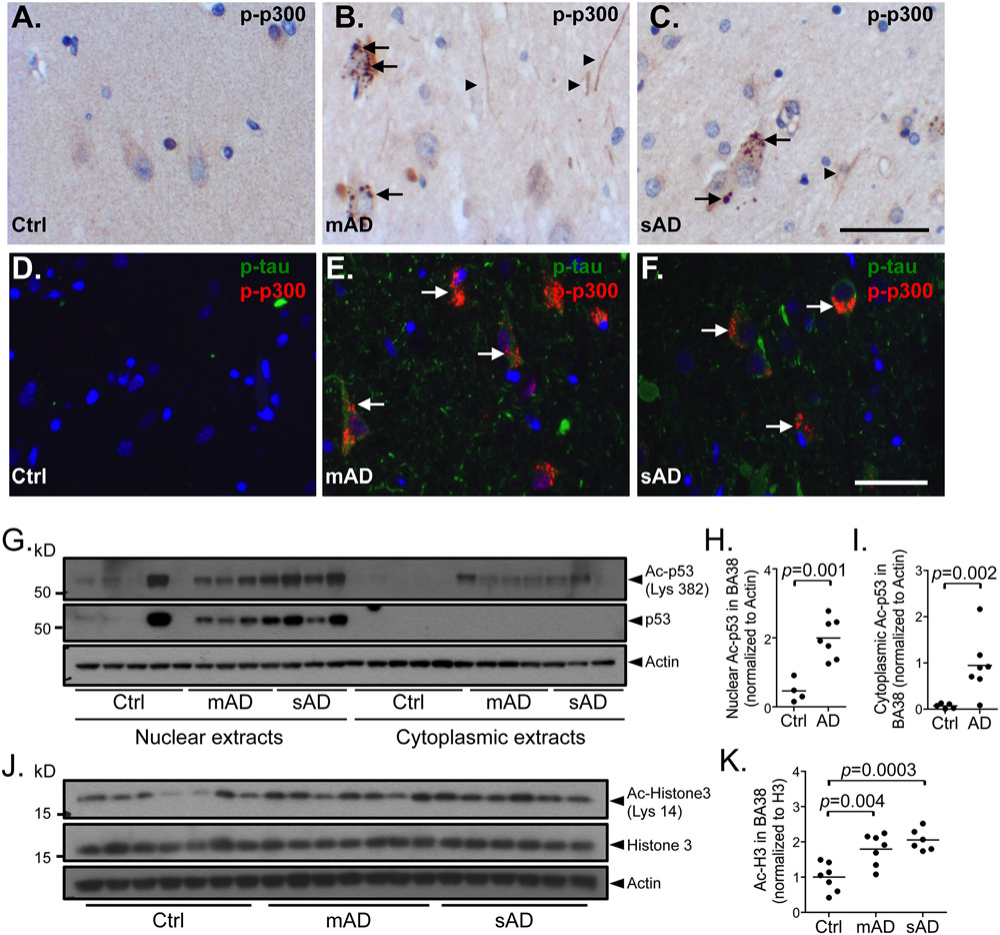
Increase in p300 acetyltransferase activity in moderate (mAD) and severe AD (sAD) cases. Immunohistochemistry and immunofluorescence analyses of phosphor-Ser1834-p300 (p-p300) on paraffin sections from the hippocampal area CA1 of human brains from control (Ctrl) (*A*, *D*), moderate AD (mAD) (*B*, *E*) and severe AD (sAD) (*C*, *F*) show cytoplasmic granular labeling resembling granulovacuolar degeneration in pyramidal neurons (*B*, *C*, *E*, *F*; arrows) in AD tissue, which co-localize with phospho-tau (p-tau) in ~95% of p-p300-positive neurons (*E*, *F*; arrows). p-p300-positive threads (*A*, *D*, arrowheads) are also observed in affected tissue. Controls are negative for p-tau and p-p300 (*A*, *D*). (*G*) Immunoblots of nuclear and cytoplasmic fractions of temporal neocortex (BA38) from Ctrl, mAD and sAD cases show an increase in Ac-Lys382-p53 (Ac-p53) in AD (*n* = 7, mAD+sAD) compared to control (*n* = 5) in both fractions. Quantification using densitometric analysis reveals a significant increase in Ac-p53 in nuclear (*H*, *p* = 0.002) and cytoplasmic (*I*, *p* = 0.001) fractions compared to controls. (*J*) Immunoblots of whole tissue extracts from BA38 from Ctrl, mAD and sAD cases also show a significant increase in Ac-Lys14-Histone 3 (Ac-H3) in mAD (*n* = 7) and sAD (*n* = 6) compared to Ctrl (*n* = 7). (*K*) Quantification reveals a statistically significant increase in Ac-H3 in mAD (*p* = 0.004) and sAD (*p* = 0.0003) compared to control. Comparisons were made using a Student’s t-test. Control case 4 is a statistical outlier, more than 3 standard deviations above the mean, and was excluded from the analysis. (*A*–*C*) Scale bar = 50 μm, (*D*–*F*) scale bar = 50 μm.

The p300 protein is a member of the p300/CREB-binding protein (CBP) coactivator family. These proteins interact with numerous transcription factors and participate in various physiological processes including proliferation, differentiation and apoptosis, by coordinating and integrating multiple cellular events to the transcriptional machinery [34,35]. Immunohistochemical staining of control and AD brains for p300 labels perisomatic granules in moderate and AD cases, but no consistent change in staining intensity or distribution is observed (S4C and S4D Fig.). Immunohistochemical staining with an antibody to phospho-Ser1834-p300 (p-p300), which is necessary for p300 acetyl-transferase activity [36], shows AD-specific staining in both CA1 and frontal cortex sections (Fig. 5A-F and S6D Fig.). Cytoplasmic structures, consistent with granulovacuolar degeneration (GVD), are observed in pyramidal neurons from affected tissues (Fig. 5B and 5C). These structures co-localize with phospho-tau (p-tau) in ~95% of p-p300-positive neurons (Fig. 5E and 5F). Many p-p300-positive fine delicate neuritic processes are also observed in affected tissue from human CA1 region as well as in other regions including the frontal cortex (S6D Fig.). Control tissues do not show these staining patterns (Fig. 5A and 5D).

To ask whether the acetyltransferase activity of p300 is altered in AD, we studied the acetylation state of two well-described p300 substrates, Histone 3 and p53 [37,38]. p300/CBP is responsible for p53 acetylation in its C-terminal domain [39–41]. Acetylation and ubiquitination processes compete for these positions with the acetylation leading to stabilization [42,43]. Densitometric analysis shows an increase of nuclear Ac-Lys382-p53 and total p53 in AD samples compared to controls in temporal neocortex (Fig. 5G and 5H) and frontal cortex (S6A and S6B Fig.). Cytoplasmic fractions also show increased Ac-p53 (Fig. 5G and 5I). These results are consistent with the stabilization of p53 in AD tissue. Similarly, Ac-Lys14-Histone 3 is also significantly increased in whole tissue extracts from AD patients in temporal neocortex (Ctrl-mAD *p* = 0.004, Ctrl-sAD *p* = 0.0003) (Fig. 5J and 5K) and frontal cortex (*p* = 0.0006) (S6A and S6C Fig.). Therefore, these experiments suggest an increase in p300 acetyltransferase activity in AD brain tissue and highlight a possible mechanism affecting p300-mediated gene regulation.

#### 3. ZMYM3

**Fig 6:**
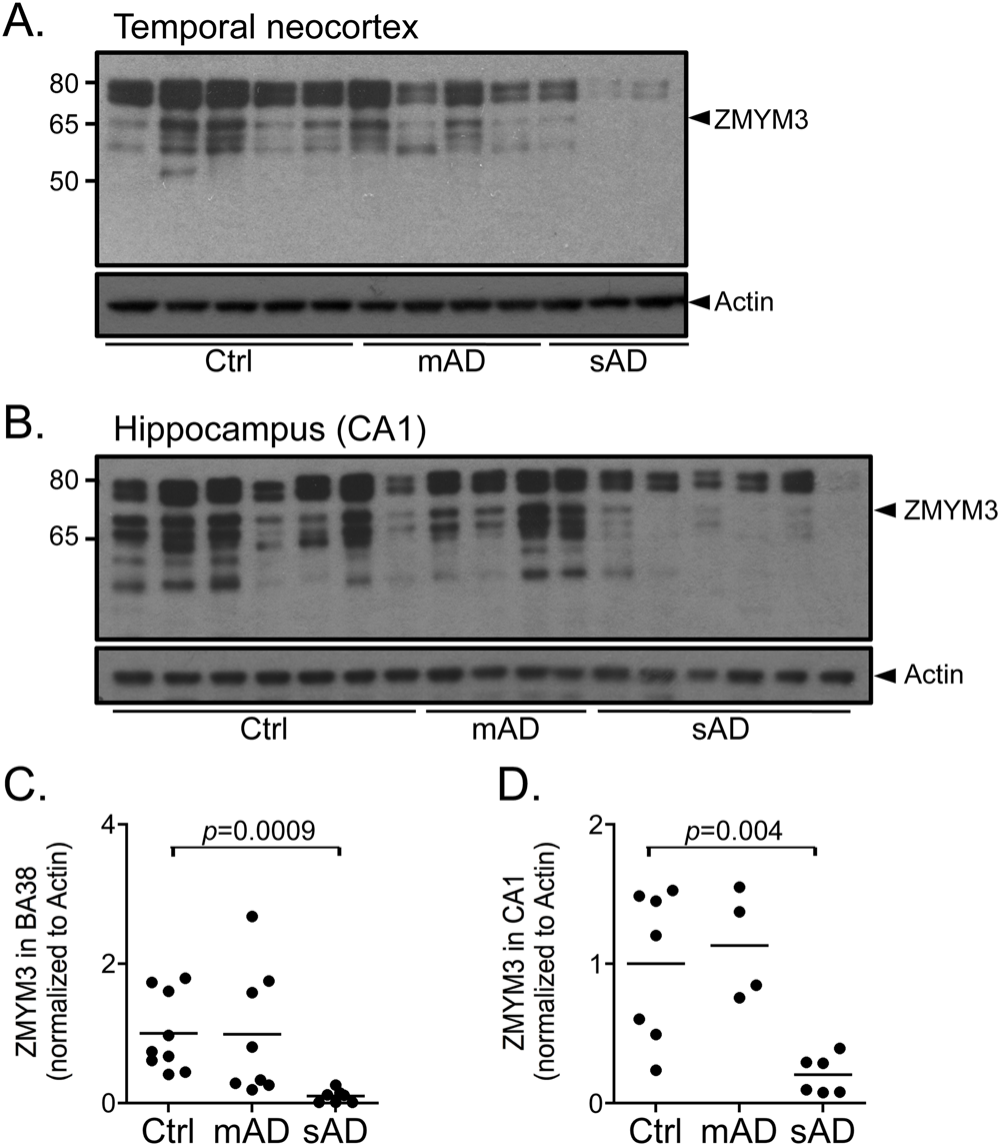
ZMYM3 protein level significantly decreases in severe AD cases. (*A*, *B*) Representative immunoblots using nuclear fractions from temporal neocortex (BA38) (*A*) and hippocampal area CA1 (*B*) show a decrease in the levels of ZMYM3 in severe Alzheimer’s disease (sAD) compared to control (Ctrl) and moderate Alzheimer’s disease (mAD). (*C*, *D*) Densitometric analyses of the 70 kDa band shows a significant decrease in ZMYM3 in severe AD cases in BA38 (*n* = 8; *p* = 0.0009) and CA1 (*n* = 6; *p* = 0.004) compared to controls in BA38 (*n* = 9) and CA1 (*n* = 7). Comparisons were made using a Student’s t-test.

ZMYM3 gene encodes a zinc-finger protein originally identified as a candidate gene for non-specific X-linked mental retardation [44]. Immunohistochemical staining of four control, four moderate AD and four severe AD cases reveals positive labeling of variable signal intensity of nuclei in a subset of neurons (S4E and S4F Fig.). Some oligodendrocytes and astrocytes are also immunopositive but no consistent difference is observed between AD and controls. Immunoblots show multiple bands with the ZMYM3 antisera, including a 70 kDa band that can be downregulated in rat primary hippocampal neurons using a ZMYM3-targeting siRNA construct (Fig. 6A and 6B and S5D Fig.). Densitometric analyses show a significant decrease in the levels of the 70 kDa ZMYM3 band in severe AD cases compared to controls in the temporal neocortex (*p* = 0.0009) and CA1 (*p* = 0.004) while moderate cases show no difference (Fig. 6C and 6D). In contrast to YY1, apoptotic concentrations of Aβ do not alter ZMYM3 levels in rat hippocampal neurons nor induce cleavage of the protein suggesting that ZMYM3 regulation is not a direct response to the altered changes of Aβ in AD (Fig. 4A). These results show that ZMYM3 levels are decreased in the nuclei of severe AD cases and Aβ treatment does not influence the protein levels at the time frames and concentrations tested.

## Discussion

Multiple pathological processes have been associated with Alzheimer’s disease (AD), highlighting the complexity of the disease. Novel computational approaches have the potential to address this complexity by enabling the interrogation of the system as a whole. Recent progress on the reliability of high-throughput technologies combined with powerful computational and statistical analyses have now rendered such approaches feasible. In this study, we apply the ARACNe and MARINa algorithms to Alzheimer’s disease and examine their utility by subsequent biochemical and histopathological analyses in human postmortem tissue. Our approach is free of *in vitro* and animal model bias and is driven by regulatory models and disease-relevant signatures obtained exclusively from the neural compartment of primary human samples, isolated by LCM. The latter avoids the significant bias due to the cellular heterogeneity that is inherent in approaches based on whole brain transcriptome profiling. Indeed, we and others have reported that transcriptional regulatory models are highly divergent in different cellular lineages and have advocated the use of context-specific regulatory models, dissected from homogenous cellular populations. Our methodology relies on the identification of candidate genes based on their inferred protein activity, rather than their expression levels, and on their direct causal relevance in implementing the observed disease-specific signatures. This represents a novel strategy in the effort to pinpoint genes whose activity drives disease progression according to various post-translational mechanisms, e.g., proteolytic regulation for YY1, post-translational modification for p300 and modulation level for ZMYM3. Two of the lead candidate MRs found by our computational approach, YY1 and ZMYM3, have not been previously implicated in AD, and our analysis has provided a number of additional candidates that we plan to investigate in follow-up studies.

The importance of this approach is heightened by the limitation of current animal models of AD, which do not replicate the full pathological picture and are therefore of limited usage for biochemical studies and drug discovery. In this regard, our Systems Biology (SB) approach provides a dynamic interpretation of the static postmortem state. Increasing the size of the dataset, by including, for example, additional tissue samples from younger AD patients would have the potential to enrich the analysis and identify candidate genes active at earlier stages of disease progression. The studies reported here were designed as “proof of principle” and we used specific assumptions and filters to identify a small subset of candidate MRs for experimental validation. This suggests that alternate filters, such as filtering by synaptic proteins rather than on brain regions will also be effective in unveiling additional candidate MRs. Finally, we wish to emphasize that the human neuronal interactome generated in this study is not specific to AD and can be interrogated using data obtained from a broad range of neurological and psychiatric diseases, allowing for the identification of neuron-specific MRs in these disorder.

The putative master regulators have interesting characteristics. YY1 is a ubiquitously expressed and highly conserved multifunctional transcription factor that can either repress or activate gene expression depending on the cellular context [45,46]. Its activity can be modulated by post-translational modifications including phosphorylation, p300/ PCAF-dependent acetylation, HDAC-dependent deacetylation, caspase-dependent cleavage, or by nucleocytoplasmic shuttling [29,47,48]. The comparison of YY1 expression in CA1 and the temporal neocortex allows the study of regions with differences in susceptibility to AD. AD arises earlier in the hippocampal formation and is more advanced there than in the adjacent temporal neocortex. The total protein YY1 signal (cleaved fragments + full protein) shows an insignificant increase in AD samples compared to controls (x1.2 fold change in BA38, unpublished data), which correlates with the increase found in YY1 mRNA levels. However, the ratio shifts to a preponderance of cleaved fragments as the pathology progresses, with almost no full-length YY1 protein detectable in CA1 nuclear fractions. The cleaved YY1 fragments are in the same molecular weight range as the N-terminally truncated YY1 fragment (YY1Δ119) previously described, which has lost its transactivation domain but retained its DNA binding domain and may act as a negative regulator [29]. Similar observations show that YY1-mediated gene regulation might be altered during neuronal degeneration because of the formation of YY1 complexes of different molecular weights [49]. Together, these results suggest an alteration of YY1 transcriptional activity in AD brain through the modulation of full-length YY1 protein levels by proteolytic activity and generation of potential dominant negative cleavage products. Additional experiments performed in a cellular Aβ toxicity model show that apoptotic concentrations of Aβ induce appearance of cleaved YY1 fragments as early as 6h following treatment, potentially positioning YY1 downstream of Aβ. The increase in full-length YY1 protein was not seen in human tissue, perhaps due to degradation. Intriguingly, YY1 has been shown in astrocytes to be an activator of BACE1, a critical protease involved in amyloidogenesis, which indicates another possible mechanism involved in AD [50]. Further experiments examining the role of YY1 in APP processing are required.

The p300/CREB-binding protein (CBP) co-activator family exerts its activity through various mechanisms. p300/CBP can facilitate protein-protein and protein-DNA interactions serving either as a protein bridge or scaffold, connecting transcription factors to the transcriptional apparatus or recruiting components to enhancer or promoter elements [51]. Alternatively, p300/CBP can exert its transcription-regulating properties through its histone acetyltransferase (HAT) activity. Our results support the hypothesis that p300 acetyltransferase activity is upregulated in AD brains, therefore modulating its transcriptional activity. Immunohistochemical analyses of human brain tissue showed a clear increase in phospho-Ser1834-p300 in AD brains while total P300 levels were unchanged. There was staining of granulovacuolar degeneration (GVD) vacuoles and neuritic processes, a subset of which are abnormal/dystrophic. Additionally, we found that p-p300 colocalizes with abnormal phospho-tau in GVD positive neurons. Intriguingly, hyperacetylation of tau has been shown to occur in AD brain and has been attributed to p300 activity, suggesting that such an interaction could occur in AD brains [52,53]. Further, we found that acetylations of p53 and Histone 3, two other prominent substrates of p300, are significantly increased in AD brain tissue compared to controls [37,38]. Interestingly, YY1 downregulation also results in p300-dependent acetylation and stabilization of p53 through the prevention of its ubiquitination [41–43,54,55]. Thus p300 and YY1 activities might converge to increase p53 levels in AD brains, which correlates with previous reports [56]. In summary, alterations in p53 may be a critical downstream effector of p300 and YY1.

Finally, ZMYM3 is highly conserved among vertebrates, ubiquitously expressed in adult tissues, but more abundant in the brain and predominantly located in the nucleus [44,57]. Our immunohistochemistry and Western blot analyses corroborate these findings and further indicate that ZMYM3 could emerge as a marker of disease severity. Indeed, despite being upregulated at the messenger RNA level, both in the original Liang at al. gene expression profiles dataset and in the QPCR analyses performed on whole tissue extracts, ZMYM3 protein levels are significantly decreased in severe AD cases, likely influencing total transcriptional activity. A compensatory mechanism counterbalancing the loss of protein might explain the increase in translational activity or ZMYM3 expression that could require another protein, as yet unidentified, providing a possible explanation for the positive NES score in the MARINa analysis. Little is known at this time about the mechanisms modulating ZMYM3 activity. Finally, we do not observe any response of ZMYM3 to Aβ treatment at the protein level: either ZMYM3 protein level is not modulated by Aβ, or the modulation is not observable at the protein level or at the tested time frames. Future studies will aim to position ZMYM3 in AD relevant pathways.

These analyses have been performed by combining the effect of different genetic backgrounds with the variability induced by the analysis of multiple brain regions. This is consistent with previous work in prostate cancer (Aytes et al. Cancer Cell 2014) where variability induced by different genetic backgrounds was combined with variability resulting from pharmacological perturbation without introducing artifacts or false positives. In the original design of the MARINa algorithm (Lefebvre et al, Mol. Systems Biol. 2010) we also addressed the issue of potential confounding issues resulting from the introduction of samples that may represent hierarchical structures in tissue stratification. Specifically, we showed that, as long as the strongly correlated samples (e.g., those coming from the same tumor subtypes) represent no more than 20% of the data, there was virtually no difference in the results of the analysis when the samples are removed from the network inference and used only in the MARINa analysis. Taken together these works support the results of this study.

Our findings also suggest that an SB approach may be a valuable addition to conventional methods for the study of neurodegenerative phenotypes, by revealing candidate MRs acting at different stages of disease progression. It is also interesting to note that two of our MRs are not affected by Aβ, suggesting that the direct analysis of human tissue will detect alterations that may not be found in the commonly used mouse models of AD. By assessing candidate MRs that are highly enriched in the hippocampus, we found MRs that are dysregulated in relatively early stages, *i*.*e*., YY1 and p300. By assessing candidate MRs that do not necessarily display a high enrichment score in the hippocampus, but are significantly ranked in the highest number of regions, we found MRs exhibiting biochemical changes in severe cases only, *i*.*e*., ZMYM3. We hypothesize that future studies analyzing expression profiles from subjects with prodromal AD or mild cognitive impairment will reveal MRs mediating the earliest stages of the disease and identify causative processes.

## Conclusions

In summary, we applied a Systems Biology approach to a set of gene expression profiles from laser-captured neurons of human brains and generated the transcriptional component of a human neuronal interactome. The interactome is by itself an invaluable resource that can be interrogated in future studies to unveil candidate MRs playing a role in other neurological contexts or at different stages of disease progression by inputting relevant gene expression profiles. Interrogating this interactome in the context of AD provided an unbiased list of candidate MRs potentially playing a role in the disease progression. We have evidence to support that three of the selected candidates exhibit dysregulated behaviors in human AD autopsy brain tissue, but further validation in cellular and animal models is necessary to understand their role in the pathology. However, these dysregulations highlight specific pathological processes that can be further studied. On one hand, both p300 and YY1 activities lead to an increased stabilization of p53, thus appearing as a potential downstream effector of both MRs and as a main player in neurodegenerative processes. On the other hand, all studied MRs are involved in HAT/HDAC activity: p300 is a HAT, YY1 activity is regulated by various HATs/HDACs such as p300 [45,58] and ZMYM3 is a component of a HDAC-containing complex [59]. These results support the hypothesis that a loss of acetylation homeostasis in AD could explain neuronal dysfunction and toxicity [60].

## Materials and Methods

### Ethics Statement

Animal work was carried out under protocols AC-AAD9106 and AAAD4910 approved by the Columbia University Institutional Animal Care and Use Committee. Rats were sacrificed using CO2 euthanasia, and all efforts were made to minimize suffering. All human tissues used for these studies were de-identified samples obtained from the New York Brain Bank under Columbia University Institutional Review Board protocol AAAB0192 (expires Feb. 2015). This study is exempt category 4 research as it involved the study of existing data and specimens recorded by the investigator in such a manner that subjects cannot be identified, directly or through identifiers linked to the subjects under OHRP Exempt Categories 45 CFT 46.101(b).

### Materials

All chemicals used were of the highest grade available. RIPA, proteases and phosphatases inhibitors were purchased from Thermo Scientific. HFIP (Hexafluoro-2-propanol) was purchased from Fluka. Poly-D-lysine, DMSO, ZMYM3 rabbit antibody (IHC, HPA003211), Actin mouse antibody (A1978) were purchased from Sigma; GAPDH mouse antibody (IMG-5019A-2) from Imgenex; Ac-Lys382-p53 rabbit antibody (2525S) and Ac-Lys14-Histone 3 rabbit antibody (4318) from Cell Signaling Technology; ZMYM3 rabbit antibody (WB, sc-130039), YY1 rabbit antibody (IHC, WB, sc-281), p300 rabbit antibody (IHC, sc-585) and p53 mouse antibody (WB, sc-126) from Santa Cruz Biotechnology; MEF2D mouse antibody (IHC, WB, 610774) from BD Biosciences; βIII-tubulin from R&D systems; phospho-Ser1834-p300 (IHC, AP3296a) from Abgent; phospho-tau (p-tau) mouse antibody (clone AT8) from Thermo Scientific. Amyloid beta (1–42) peptide was ordered from UCLA.

### Patient samples

Fresh frozen and formalin fixed/paraffin-embedded autopsy brain tissue were obtained from the New York Brain Bank at Columbia University Medical Center (New York, NY, USA) for immunoblot and immunohistochemistry. Neuropathological examination was per the brain bank protocols [61]. Samples were classified as control, moderate AD (mAD) or severe AD (sAD) as presented in S8 Table. When no significant difference was observed between mAD and sAD, measurements were pooled for statistical analyses.

### Dataset Processing and Normalization

The analysis was performed on the Liang et al. dataset [20,21]. The dataset was downloaded from the Gene Expression Omnibus website, reference GSE5281 and GSE9770, and contained 193 samples corresponding to six regions obtained from 14 controls, 10 NDAD individuals and 34 demented individuals with the histopathological confirmation of AD. The gene expression profiles in the dataset were collected using the Affymetrix Human Genome U133 Plus 2.0 Array GeneChip system (54,675 probe sets). Expression measurements were normalized with gcrma [62], which adjusts for background intensities in Affymetrix array data, including adjusting for optical noise and non-specific binding. The array files were processed and normalized using R version 2.15.1.

### Hierarchical Clustering

Clustering analysis was performed using BRB-Array tools version 4.3.2. Out of the 54,675 probesets that were present on the array, 23,594 probesets that passed the BRB-Array tools filters were used to perform the clustering. The arrays were normalized using a modified version of RMA, which uses a random subset of the arrays to generate the normalization and probset summary values. Centered (Pearson) correlation was used as the distance metric, and the average distances between all pairs of probesets that had an associated p-value of < 0.01 were used to build the clusters.

### ARACNe

Context specificity is critical to reconstruct the transcriptional regulatory logic as TF regulons are highly context dependent. The ability to accurately reconstruct such logic using ARACNe is predicated on the availability of large gene expression profiles datasets, including more than 100 samples, which represent either natural or perturbation-induced genomic variability of the context of interest. For the reconstruction of the human neuronal interactome, we assumed that different individuals represented sufficient naturally occurring genotypic variability, while the different regions represented sufficient variability associated with microenvironment related signals, thus resulting in an interactome representing an accurate estimate of the overall variability of cortical neuron gene expression. This assumption is supported by the t-SNE [23] results on all samples, which show separation between regions that are severely affected in AD, as well as high variability between regions overall (S2 Fig.). Thus, in contrast to traditional approaches, where one attempts to minimize variability by profiling several biological replicates, ARACNe benefits from inter-sample variability as this allows for a more accurate inference of transcriptional interactions by taking into account inter-sample transcriptional regulatory differences. In contrast, the signatures used to interrogate the interactome require significant phenotypic specificity and benefit from replicate samples.

To construct the neuronal transcriptional network, ARACNe was applied to the set of 193 gcrma-normalized expression profiles using the adaptive partitioning algorithm, which selects the optimal kernel width for calculating the Mutual Information (MI) threshold of a specified p-value. The MI threshold used by ARACNe (MI > = 0.2185) corresponded to the p-value threshold of 10^-7^ after 100 bootstrap runs. The resulting central nervous system (CNS) Interactome contained 488,353 statistically significant MIs between the 3,758 TFs and 38,045 genes.

### MARINa and Candidates Selection

MARINa was used to infer MRs that drive the transition between Control and AD samples, as defined by pathological and clinical characterization. Given a regulatory network model, MARINa requires a relatively small numbers of gene expression profiles (*N* ≥ 6) representing each phenotype of interest in a specific transition (e.g., 6 gene expression profiles for NDAD and 6 for AD), to identify the candidate MR genes. For each phenotype transition of interest (*e*.*g*., Control → AD in HIP), we first generate the gene expression profile signature, defined as all genes represented on the specific microarray platform, ranked from the most underexpressed to the most overexpressed in the disease phenotype compared to Control samples, as determined by a t-test. MRs are then identified as the TFs with the highest likelihood of implementing the specific signature based on the regulatory mode, i.e., those whose ARACNe-inferred targets (i.e. TF-regulon) are most enriched in differentially expressed genes in the gene expression profile signature. For instance, a positive MR (whose activation drives the transition) would have its positively regulated targets highly enriched in overexpressed genes and repressed targets highly enriched in under expressed genes in the signature of interest. The opposite would be true for negative MRs (whose inactivation drives the transition). Such enrichments can be effectively assessed by a multi-tail extension of the Gene Set Enrichment Analysis (GSEA) [24]. Thus, importantly, identification of candidate MRs is based on the expression of their ARACNe-inferred targets rather than on their own change in expression level.

Master Regulator INference analysis (MARINa) was used to analyze each TF with more than 20 targets in the human neuronal transcriptional network, with GSEA [24] used to assess the enrichment of each TF’s set of predicted targets. As a reference, we used the set of genes on the expression profile, ranked by their t-statistic calculated by comparing the candidate phenotypic transition of interest (*e*.*g*., Control → AD in HIP). P-values were computed by performing 1,000 sample-shuffling permutations and each TF was given a p-value based on its enrichment score according to GSEA. Shadow Analysis was performed as described in Lefebvre et al [16], to eliminate false positive representing TFs with substantially overlapping programs with bona fide MRs but unlikely to drive the signatures of interest. Master Regulator candidates (i.e., TFs with p < 0.01, not removed by Shadow Analysis) were sorted by their Differentially Expressed Targets Odds Ratio (DETOR) score, as given by Master Regulator Analysis [16]. The counts of candidate MRs obtained in each region at this step can be found in Fig. 2.

### Transcription Factors classification

To identify transcription factors (TFs), we selected the mouse genes annotated as “transcription factor activity” in Gene Ontology and the list of TFs from TRANSFAC. This produced a final list of 1,794 TFs, which mapped to 3,758 probesets on the gcrma-normalized expression profile.

### Specificity-weighted GSEA and Bootstrapping

To further classify the MRs that were most relevant for disease outcome, we applied specificity-weighted GSEA in combination with bootstrapping of the samples. Specificity-weighted GSEA is an alternative method that can be used to predict master regulator candidates. Similar to MARINa, specificity-weighted GSEA begins by measuring the enrichment of differentially expressed targets for each TF. However, in calculating the enrichment score for each TF and its target, it takes into account the specificity of interaction between TF and its target, i.e. the total number of TFs regulating the target according to the neuronal transcriptional network.

In the original GSEA paper by Subramanian [24] describes the calculation of the Enrichment Score (ES) in the following way, with ES being the maximum deviation from zero of *P*_*hit*_—*P*_*miss*_.

- Rank order *N* genes on the microarray platform to form *L = g*_*1*_ … *g*_*N*_ according to the t-score of their differential expression between phenotype 1 and phenotype 2
- For each TF being tested by specificity-weighted GSEA, calculate the fraction of genes in S (“hit”, or targets of the TF according to the neuronal transcriptional network) weighted by their correlation and the fraction of genes not in S (“miss”) present up to a given position *i* in *L*. where

$$P_{hit}\left(S,i\right)=\;\sum_{j\leq i}^{g_{j}\in \;S}\frac{{\left|r_{j}\right|}^{p}}{N_{R}}$$

$$N_{R}=\;{\sum_{g_{j}\notin \;S}\left|r_{j}\right|}^{p}$$

$$P_{miss}\left(S,i\right)=\;\sum_{j\leq i}^{g_{j}\notin \;S}\frac{1}{N-N_{H}}$$

In specificity-weighted GSEA, the “hit” score of each gene is calculated in the following way,

$$P_{weighted-hit}\left(S,i\right)=\;\sum_{j\leq i}^{g_{j}\in \;S}\frac{{\left|r_{j}\right|}^{p}}{N_{R}T_{j}}$$

Where T_j_ is the number of TFs that regulate gene R_j_. The application of this equation results in more conservative estimate of the enrichment score of each TF. In addition, the method rewards TFs that have specific targets.

In order to select for highly enriched MR candidates, 10 iterations of specificity-weighted GSEA was run with bootstrapping. Each bootstrap run was performed using 70% of samples in each class (random subset with replacement) and 1,000 sample-shuffling permutations. Each TF was given a p-value based on its enrichment score according to specificity-weighted GSEA, and the results were sorted by their DETOR score [16]. Eventually we only selected MR candidates that had a p-value < 0.01 according to specificity weighted GSEA in 10 out of 10 bootstrap runs. The use of bootstrapping and specificity-weighted GSEA allowed us to identify two groups of candidate MRs: those whose predicted activities were highly enriched regardless of which individual samples were used ("stable” candidates), and another group whose predicted activities showed significant fluctuations depending on which individual samples were used (“unstable” candidates). Number of stable candidate MRs in each of the regions and comparisons, as well as their effect on the final results can be found in Fig. 2.

### Quantitative real-time polymerase chain reaction (QPCR)

Fresh-frozen brain tissue was pulverized in liquid nitrogen, lysed in QIAzol and homogenized using a Qiagen TissueLyser II with 5mm stainless steel beads (frequency 25/s, 2 x 2min). RNA was extracted using an RNeasy Mini kit (Qiagen) and RNA integrity was assessed using a bioanalyzer (Agilent 2100). Only samples showing a RNA Integrity Number (RIN) score above 5 were used for further experiments. cDNA synthesis was performed using a First Strand cDNA Synthesis Kit (Origene, Rockville, MD, USA) and used as template (1:4 dilution) in 20 μl reactions. Primers sequences used for the RT-PCR were the following:

*p300: F: AGATGGGAATGATGAACAACC, R: ACTCACCATGTTGGGCATTC;*

*YY1: F: GCGGAGCCCTCAGCCATGGCCTCG, R: CAGCGGCTGCAGAGCGATCATGG;*

*ZMYM3: F: TGTGGATCGTAATGGCAAGA, R: TGCGGTCAACCTTGTTGTAG,*

*MEF2D: F: TACCCACAGCACCCAGCTT, R: TAGACTGGGAGACCCAAGG*.

The FastStart Universal SYBR Green Master mix (Roche) was used for reactions on a Mastercycler ep realplex (Eppendorf, Hauppauge, NY, USA). The mRNA levels were normalized against the geometric mean of GAPDH, HPRT1, SDHA and UBC as described in Vandesompele et al. [63].

### Protein extraction from whole tissue

Approximately 50 mg of fresh-frozen brain tissue were pulverized in liquid nitrogen, dounce homogenized in ice-cold PBS, diluted in RIPA buffer containing proteases and phosphatases inhibitors, sonicated, centrifuged and aliquoted to be stored at -80°C. Concentrations were measured using a BCA assay and samples were diluted to 1 mg/ml. For nuclear-cytoplasmic extractions, approximately 80 mg of fresh-frozen brain tissue were pulverized in liquid nitrogen, washed once with ice-cold PBS and centrifuged at 500 g for 5 min at 4°C. The supernatant was discarded and the pellet was resuspended in 700 μl of 1x Hypotonic buffer, containing proteases and phosphatases inhibitors, and incubated on ice for 15 min. Samples were then dounce homogenized and reincubated on ice for 15 min. 35 μl of 10% NP40 were added, samples were vortexed for 10 seconds at highest setting and incubated on ice for an additional 5 min. The homogenate was centrifuged for 10 min at 14,000 g at 4°C. The supernatant contains the cytoplasmic fraction and was aliquoted and stored at -80°C. The nuclear pellet was resuspended in 50 μl of Buffer B Working reagent from Procarta Nuclear Extraction Kit (Affymetrix, cat # AY2002) and the protocol was followed according to instructions. Nuclear extracts were aliquoted and stored at -80°C. Concentrations of nuclear and cytoplasmic extracts were measured using Bio-Rad DC Protein Assay kit.

### Immunohistochemistry

The slides were deparaffinized in xylene, rehydrated through graded alcohol and antigen retrieved by pressure cooking for 30 minutes in ethylenediamine tetraacetic acid (EDTA) buffer for MEF2D; in citrate buffer for p300, phospho-Ser1834-p300 and YY1 and in Trilogy for ZMYM3. The slides were processed following the EnVision + Dual Link Kit (Dako) and incubated overnight at 4°C with rabbit polyclonal anti-p300 (Santa Cruz, 1:50), rabbit polyclonal anti-p-p300 (1:30), rabbit polyclonal anti-YY1 (1:50), rabbit polyclonal anti-ZMYM3 (1:800), mouse monoclonal anti-MEF2D (1:100) diluted in Dako antibody diluent. The slides were stained with DAB (3,3-diaminobenzidine) as described in the protocol and counterstained with Mayer’s hematoxylin, dehydrated, and mounted.

### Immunocytochemistry

The slides were deparaffinized in xylene, rehydrated through graded alcohol and antigen retrieved by pressure cooking for 30 minutes in citrate buffer for 30 min. The slides were blocked 30 min using a solution of 0.1% Triton in Superblock blocking buffer (Thermo Scientific). Rabbit p-p300 (1:30) and mouse p-tau (1:100) were incubated overnight at 4°C in Dako antibody diluent. The slides were washed and goat anti-mouse and anti-rabbit antibodies diluted in Dako antibody diluent were added for 30 min. After three final washes, the slides were incubated 5 min in Sudan Black, washed again in PBS and mounted. Fluorescent images were taken with Axiovision software through Axiophot camera and a Zeiss Axioplan 2 microscope.

### Hippocampal neuron cultures and treatment

Hippocampal neuron cultures from both male and female rats pups were prepared following a slightly modified version of the method of Brewer (Brewer et al., 1993). Hippocampal neurons were kept in culture at 37°C with 5% CO_2_ in Neurobasal medium (Invitrogen) with B27 supplement and Glutamax (Invitrogen) and plated at a density of 2.5x10^5^ cell/ml on dishes coated with poly-D-lysine. For our experiments, neurons were treated after approximately 7–14 days *in vitro* (DIV) with a dodecameric preparation of Aβ_1–42_ prepared according to Barghorn et al. method [64]. At the end of the treatment, cells were washed twice with ice-cold PBS, harvested with RIPA buffer containing phosphatases and proteases inhibitors, sonicated and centrifuged.

### Western blotting analysis

Equal amounts, ranging from 1 to 10 μg, of cell and whole tissue lysates were resolved by SDS-polyacrylamide gel electrophoresis (4–12% Bis-Tris, Invitrogen) and electro-transferred on a polyvinylidene difluoride (PVDF) membrane (Millipore). The membranes were then incubated for 1h at RT in 5% milk and overnight at 4°C in primary antibodies (ZMYM3 1:100; YY1 1:100; Ac-p53 1:1,000; p53 1:100; Ac-Histone 3 1:1,000; Histone 3 1:1,000; Actin 1:5,000; GAPDH 1:5,000). For the competitive assay, two identical gels were run using 5 to 10 μg protein extracts from different human post-mortem brain regions (CA1, BA38, BA9) and from rat hippocampal neurons. The blots were then incubated overnight in 0.5 μg of YY1 primary antibody (sc-281) containing, or not, 80x of blocking peptide (sc-281 P). The antibody, with and without peptide, was pre-incubated 2h at RT in 500 μL of PBS before being diluted and applied onto the membrane. The immunoreactive signals were detected with enhanced chemiluminescence kit (Amersham Biosciences, Uppsala, Sweden). The procedures followed were conducted in accordance with the manufacturer’s instructions.

### Pen-siZMYM3 synthesis

A 5’ thiol-modified siZMYM3 (GGAGTCTCCTCATATTGAA, Dharmacon) was reduced following Dharmacon protocol. The last precipitation step was repeated three times in order to fully eliminate the excess TCEP. The dry pellet was then reconstituted in sterile annealing buffer to 90 μM. The desired amount of reduced siZMYM3 was incubated with 1 equivalent of activated penetratin (MP biomedical, cat # 11PENA0500) for 1h at 37°C and analyzed using a 20% TBE gel electrophoresis. The conjugated pen-siZMYM3 was aliquoted and stored at -80°C.

## Supporting Information

S1 Fig Dendrograms showing clustering of samples for (*A*) enthorinal cortex, (*B*) middle temporal gyrus, (*C*) posterior cingulate and (*D*) superior frontal gyrus using centered correlation and average linkage. C = control; NDAD = non-demented Alzheimer’s disease; AD = Alzheimer’s disease.
(PDF)

S2 Fig The results of t-SNE on the principle components of gene expression in the AD dataset. Prior to applying the t-SNE method, principle component analysis was performed on the expression data to extract the most informative features. The first 20 principle components were used to run t-SNE. As the plot shows, regions severely affected in AD, such as EC and HIP, show separation according to phenotype, whereas the rest of the samples show more variability. These results supported our assumption that the samples in the dataset exhibited the variability we needed to be able to accurately reconstruct the neuronal interactome using ARACNe.
(PDF)

S3 Fig Fold changes of mRNA levels between Alzheimer’s disease (AD) and controls are similar when comparing data from laser-captured neurons in middle temporal gyrus (MTG) and whole tissue extracts from the temporal neocortex (BA38). The Q-PCR analysis performed on whole tissue extracts from AD (*n* = 11) and controls (*n* = 8) shows a significant increase in mRNA level for *YY1* (*p* = 0.04) and *ZMYM3* (*p* = 0.009), a non-significant increase for *EP300* (*p* = 0.06) and a stable level for *MEF2D*, which are comparable to those observed in the laser-captured neuron dataset. Statistical analyses were performed using the Student’s t-test.
(PDF)

S4 Fig Immunohistochemistry of YY1, total p300 and ZMYM3 proteins in paraffin-embedded brain sections isolated from the CA1 region of post-mortem controls (Ctrl) and moderate Alzheimer’s disease (mAD) subjects. (*A*-*B*) YY1 staining shows that most neurons are labeled in the cytoplasm with a weak variable reticular staining in the nucleus. Amyloid plaques are variably positive, predominantly in the central core (not shown). Numerous reactive astrocytes exhibit cytoplasmic staining. However the high variability in immunostaining between cases precludes distinguishing Alzheimer’s disease (AD) from controls in a reliable manner. (*C*-*D*) Total p300 staining shows cytoplasmic granules (arrowheads) that are found in moderate and severe AD cases, but no major change in intensity or distribution pattern is observed. (*E*-*F*) ZMYM3 staining shows immunopositivity in the nuclei of a subset of neurons as well as of some oligodendrocytes and astrocytes. Scale bar = 100μm.
(PDF)

S5 Fig (*A*) Endogenous YY1 can be cleaved by caspases 3, 7, 8 and 9. Rat hippocampal neurons extracts are incubated with recombinant caspases 2, 3, 6, 7, 8 and 9 for 1hr at 37°C and analyzed by Western blot. Caspases 3 and 7 are able to hydrolyze YY1 into fragments of 35 kDa, caspase 8 into a 33 kDa fragment and caspase 9 into a 25 kDa fragment. (*B*) YY1 60 and 65 kDa isoforms show different cellular distribution in rat hippocampal neurons. Nuclear and cytoplasmic fractions of rat hippocampal neurons are analyzed by Western blot. The immunoblot reveals an enrichment of the 65 kDa form in the cytoplasm (Cyto) and an enrichment of the 60 kDa form in the nucleus (Nucl). (*C*) YY1 immunoreactive signals are specific. A competitive experiment using ± 80x of blocking peptide shows that all the bands detected by YY1 antibody are specific. (*D*) Identification of the 70 kDa band as ZMYM3. Rat hippocampal neurons are treated with 1 nM pen-siZMYM3 for two, five and seven days and analyzed by Western blot. The immunoblot reveals that the 70 kDa band is downregulated.
(PDF)

S6 Fig Increase in p300 acetyltransferase activity in the frontal cortex of Alzheimer’s disease (AD) patients. (A) Immunoblots of human brain whole tissue extracts (BA9) from controls (Ctrl) and ADs show an increase in Ac-Lys382-p53 (Ac-p53) and Ac-Histone 3 (Ac-H3) in AD cases (*n* = 7, mAD+sAD) compared to controls (*n* = 7). (*B*-*C*) Quantification reveals a significant increase for Ac-p53 (*p* = 0.04) and Ac-H3 (*p* = 0.0006). (*D*) Immunohistochemistry of p-p300 on paraffin-embedded sections from the frontal cortex (BA9) of AD shows p-p300-positive dystrophic processes. Comparisons were made using the Student’s t-test. Scale bar = 100 μm.
(PDF)

S1 Table Summary of the number of gene expression profiles used for computational analysis based on clinical and pathological classification (PDF)

S2 Table List of candidate MRs for each comparison (Control versus NDAD, Control versus AD, and NDAD versus AD) in HIP after bootstrap and overlap. (PDF)

S3 Table List of candidate MRs for each comparison (Control versus NDAD, Control versus AD, and NDAD versus AD) in EC after bootstrap and overlap. (PDF)

S4 Table List of candidate MRs for each comparison (Control versus NDAD, Control versus AD, and NDAD versus AD) in PC after bootstrap and overlap. (PDF)

S5 Table List of candidate MRs for each comparison (Control versus NDAD, Control versus AD, and NDAD versus AD) in MTG after bootstrap and overlap. (PDF)

S6 Table List of candidate MRs for each comparison (Control versus NDAD, Control versus AD, and NDAD versus AD) in SFG after bootstrap and overlap. (PDF)

S7 Table List of candidate MRs for each comparison (Control versus NDAD, Control versus AD, and NDAD versus AD) in VCX after bootstrap and overlap. (PDF)

S8 Table Summary of brain tissue used for immunoblots and immunohistochemistry. (PDF)
