# Supplementary figures and images for: Assembly and Interrogation of Alzheimer’s Disease Genetic Networks Reveal Novel Regulators of Progression
Source: PLoS One. 2015 Mar 17;10(3):e0120352. doi: 10.1371/journal.pone.0120352 (PMC4363671; doi:10.1371/journal.pone.0120352)

A.

## Entorhinal Cortex

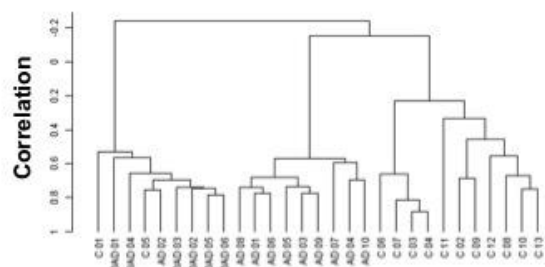

B.

## Middle Temporal Gyrus

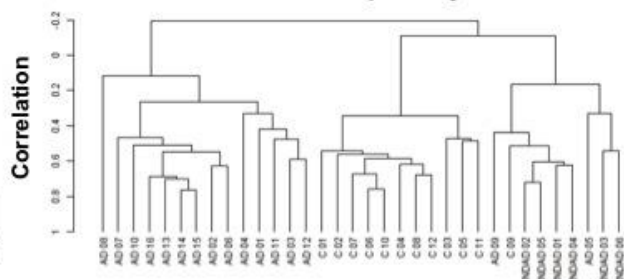

C.

## Posterior Cingulate

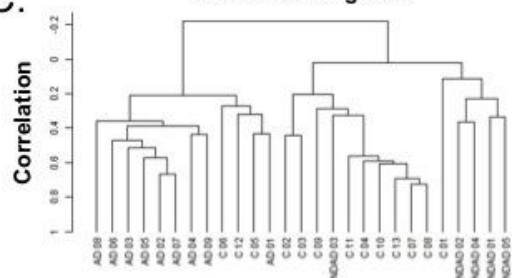

D.

## Superior Frontal Gyrus

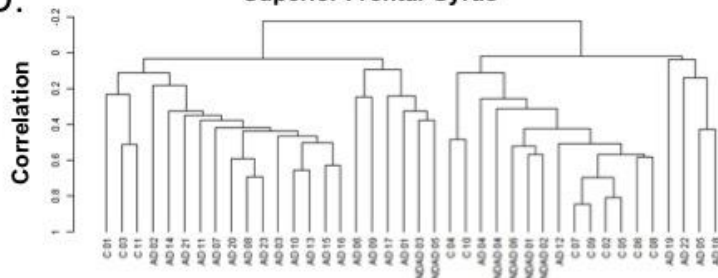

Supplement: S1 Fig — C = control; NDAD = non-demented Alzheimer’s disease; AD = Alzheimer’s disease. (PDF) [file pone.0120352.s001.pdf]

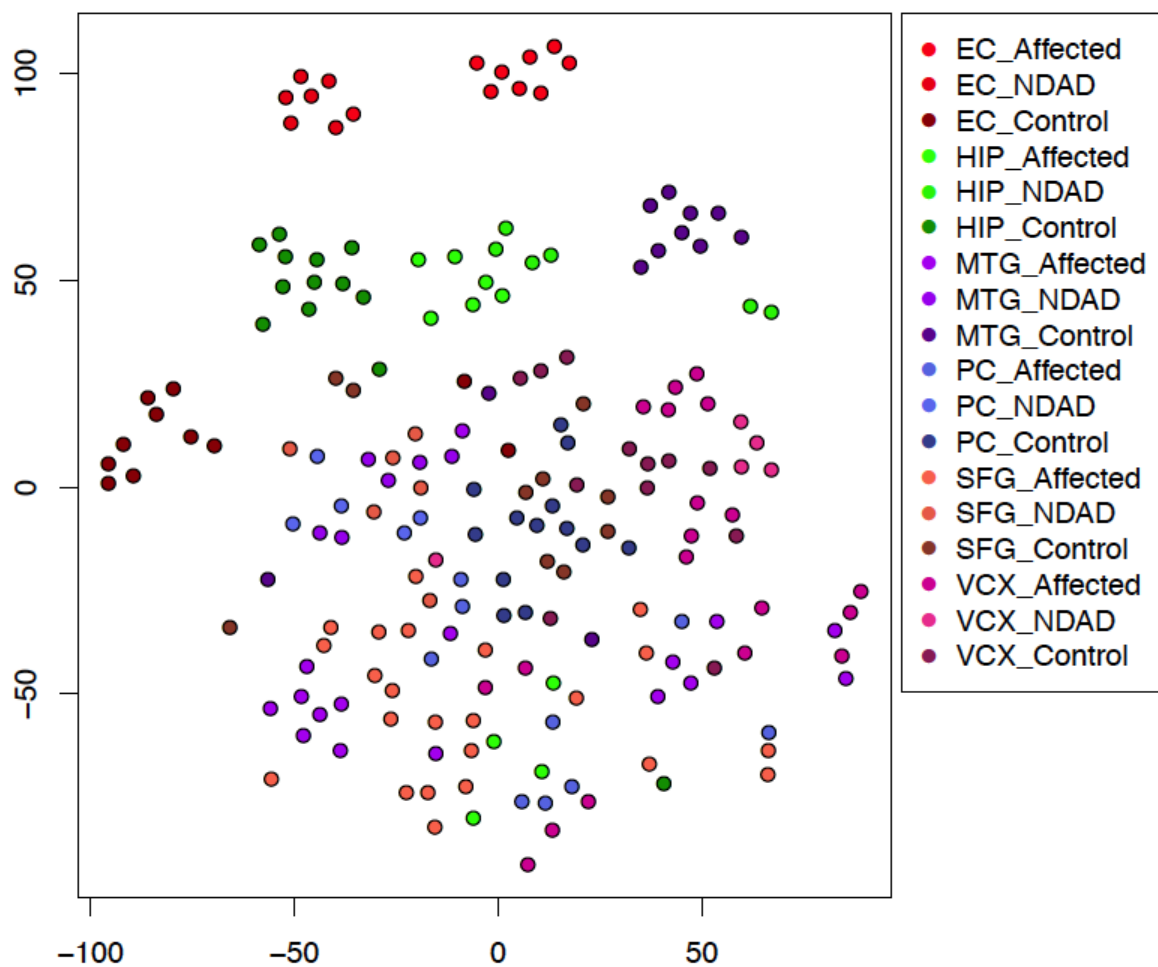

Supplement: S2 Fig — Prior to applying the t-SNE method, principle component analysis was performed on the expression data to extract the most informative features. The first 20 principle components were used to run t-SNE. As the plot shows, regions severely affected in AD, such as EC and HIP, show separation according to phenotype, whereas the rest of the samples show more variability. These results supported our assumption that the samples in the dataset exhibited the variability we needed to be able to accurately reconstruct the neuronal interactome using ARACNe. (PDF) [file pone.0120352.s002.pdf]

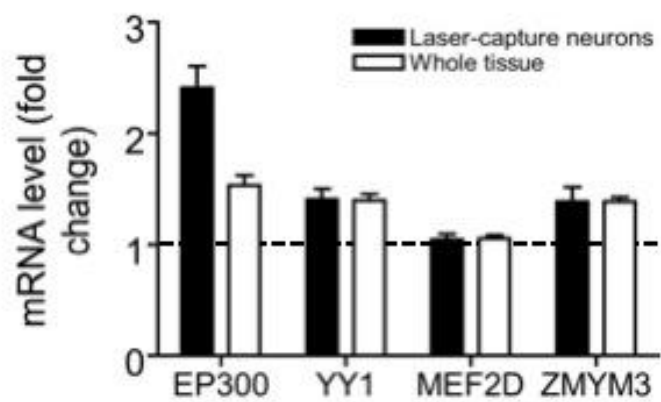

Supplement: S3 Fig — The Q-PCR analysis performed on whole tissue extracts from AD (n = 11) and controls (n = 8) shows a significant increase in mRNA level for YY1 (p = 0.04) and ZMYM3 (p = 0.009), a non-significant increase for EP300 (p = 0.06) and a stable level for MEF2D, which are comparable to those observed in the laser-captured neuron dataset. Statistical analyses were performed using the Student’s t-test. (PDF) [file pone.0120352.s003.pdf]

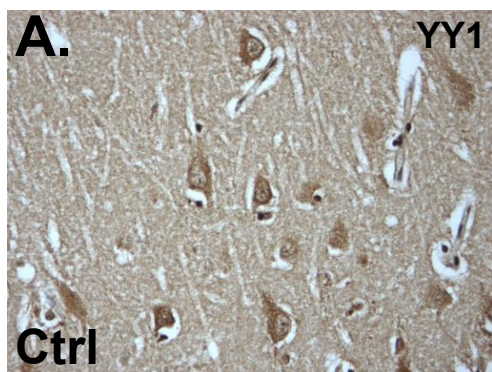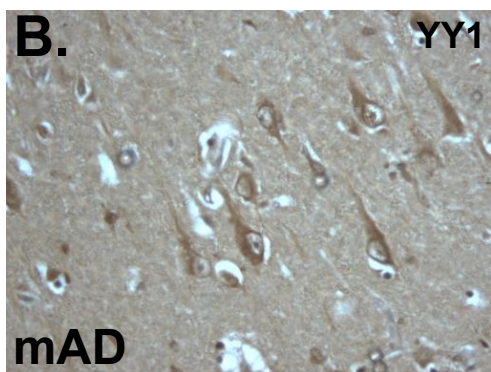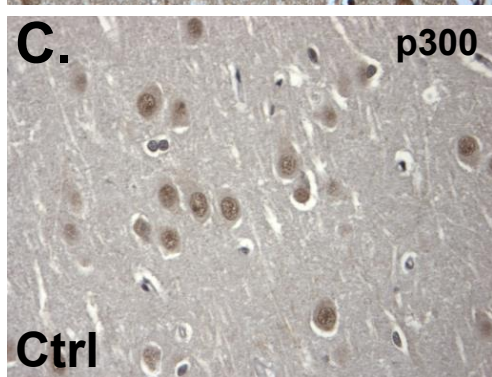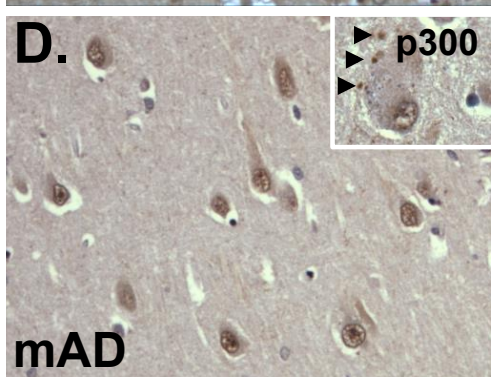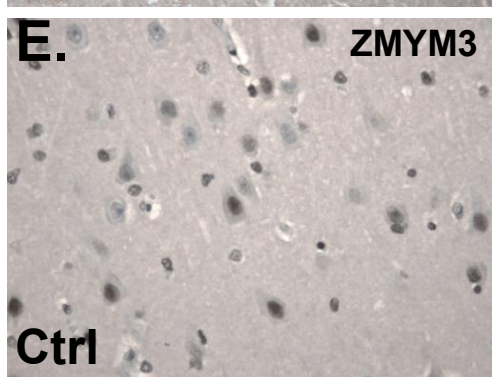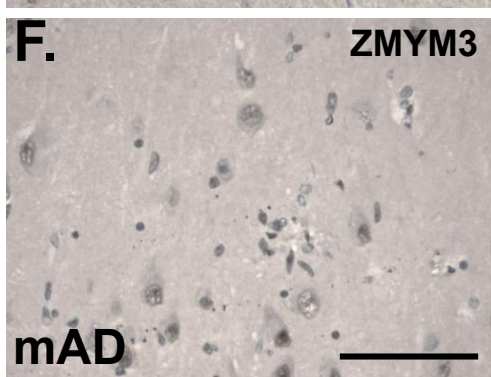

Supplement: S4 Fig — (A-B) YY1 staining shows that most neurons are labeled in the cytoplasm with a weak variable reticular staining in the nucleus. Amyloid plaques are variably positive, predominantly in the central core (not shown). Numerous reactive astrocytes exhibit cytoplasmic staining. However the high variability in immunostaining between cases precludes distinguishing Alzheimer’s disease (AD) from controls in a reliable manner. (C-D) Total p300 staining shows cytoplasmic granules (arrowheads) that are found in moderate and severe AD cases, but no major change in intensity or distribution pattern is observed. (E-F) ZMYM3 staining shows immunopositivity in the nuclei of a subset of neurons as well as of some oligodendrocytes and astrocytes. Scale bar = 100μm. (PDF) [file pone.0120352.s004.pdf]

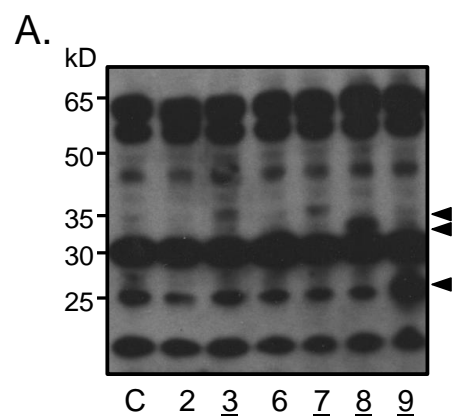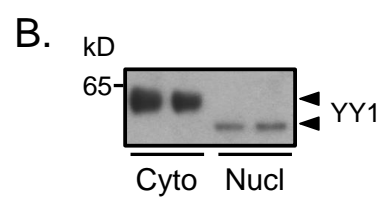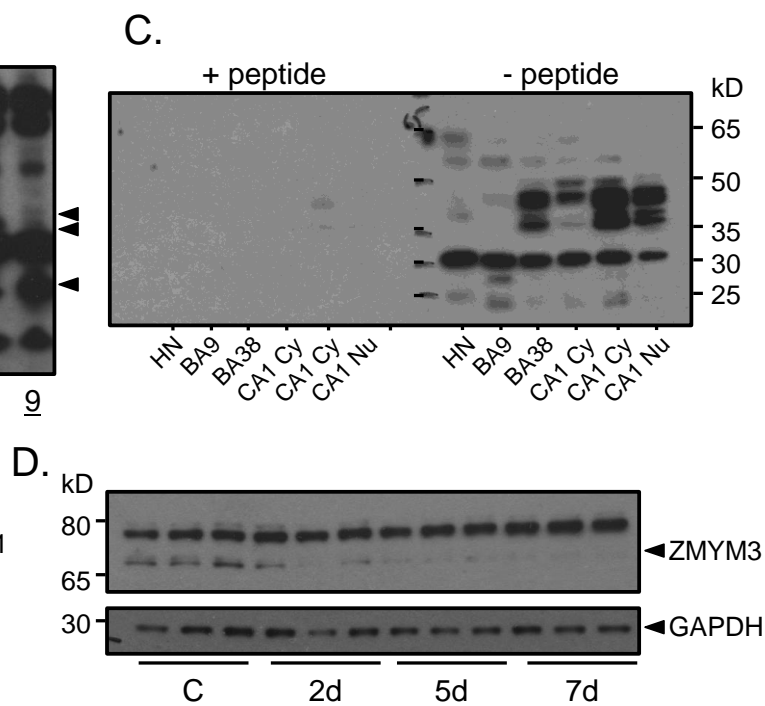

Supplement: S5 Fig — Rat hippocampal neurons extracts are incubated with recombinant caspases 2, 3, 6, 7, 8 and 9 for 1hr at 37°C and analyzed by Western blot. Caspases 3 and 7 are able to hydrolyze YY1 into fragments of 35 kDa, caspase 8 into a 33 kDa fragment and caspase 9 into a 25 kDa fragment. (B) YY1 60 and 65 kDa isoforms show different cellular distribution in rat hippocampal neurons. Nuclear and cytoplasmic fractions of rat hippocampal neurons are analyzed by Western blot. The immunoblot reveals an enrichment of the 65 kDa form in the cytoplasm (Cyto) and an enrichment of the 60 kDa form in the nucleus (Nucl). (C) YY1 immunoreactive signals are specific. A competitive experiment using ± 80x of blocking peptide shows that all the bands detected by YY1 antibody are specific. (D) Identification of the 70 kDa band as ZMYM3. Rat hippocampal neurons are treated with 1 nM pen-siZMYM3 for two, five and seven days and analyzed by Western blot. The immunoblot reveals that the 70 kDa band is downregulated. (PDF) [file pone.0120352.s005.pdf]

A.

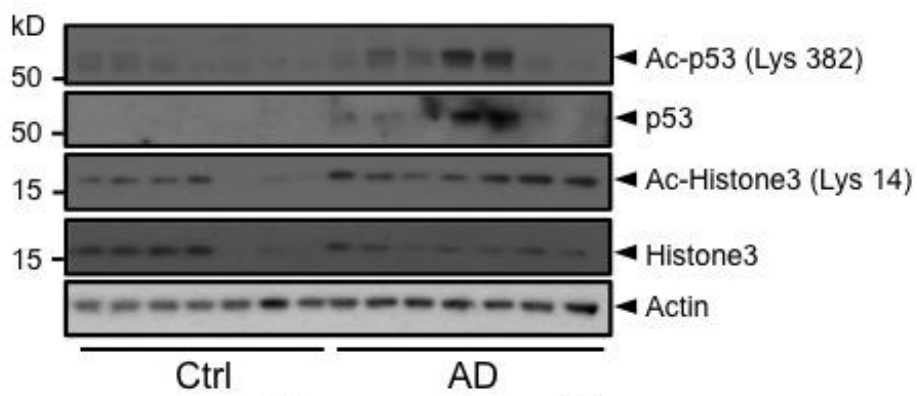

B.

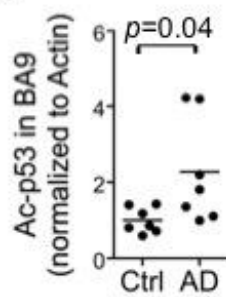

C.

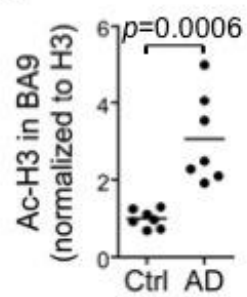

D.

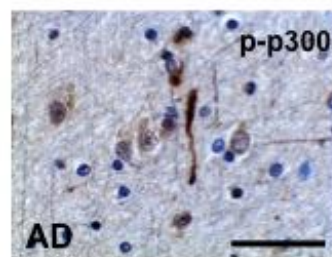

Supplement: S6 Fig — (A) Immunoblots of human brain whole tissue extracts (BA9) from controls (Ctrl) and ADs show an increase in Ac-Lys382-p53 (Ac-p53) and Ac-Histone 3 (Ac-H3) in AD cases (n = 7, mAD+sAD) compared to controls (n = 7). (B-C) Quantification reveals a significant increase for Ac-p53 (p = 0.04) and Ac-H3 (p = 0.0006). (D) Immunohistochemistry of p-p300 on paraffin-embedded sections from the frontal cortex (BA9) of AD shows p-p300-positive dystrophic processes. Comparisons were made using the Student’s t-test. Scale bar = 100 μm. (PDF) [file pone.0120352.s006.pdf]
