# Supplementary material for: Assembly and Interrogation of Alzheimer’s Disease Genetic Networks Reveal Novel Regulators of Progression
Source: PLoS One. 2015 Mar 17;10(3):e0120352. doi: 10.1371/journal.pone.0120352 (PMC4363671; doi:10.1371/journal.pone.0120352)
Supplement: S1 Table — (PDF) [file pone.0120352.s007.pdf]

	**Control**	**NDAD**	**AD**
Age of death (yr)	79.8 ± 9.1	86.6 ± 5.3	79.9 ± 6.9
Braak Stage	I - II	II - IV	V - VI
CERAD (neuritic plaque density)	Infrequent	Moderate / frequent	Moderate / frequent
Clinical diagnostic	Non-demented	Non-demented	Demented
EC	11	6	9
HIP	13	6	10
MTG	11	6	16
PC	13	5	9
SFG	10	6	23
VCX	12	5	19

NDAD non-demented Alzheimer's disease, *AD* Alzheimer's disease, *CERAD* consortium to establish a registry for Alzheimer's disease, *EC* entorhinal cortex, *HIP* hippocampus, *MTG* middle temporal gyrus, *PC* posterior cingulate, *SFG* superior frontal gyrus, *VCX* visual cortex (Liang et al, 2008a; Liang et al, 2010).
