# Supplementary material for: Assembly and Interrogation of Alzheimer’s Disease Genetic Networks Reveal Novel Regulators of Progression
Source: PLoS One. 2015 Mar 17;10(3):e0120352. doi: 10.1371/journal.pone.0120352 (PMC4363671; doi:10.1371/journal.pone.0120352)
Supplement: S2 Table — (PDF) [file pone.0120352.s008.pdf]

Gene Name	Probe Number	NES	Odds Ratio	Additional Regions
Control versus Affected				
KDM5A	226367_at	2.092	712.302	MTG
ZNF131	221842_s_at	2.002	278.485	MTG
PHF3	217951_s_at	1.997	216.843	MTG
UBN1	209088_s_at	2.008	171.193	MTG
ZNF510	206053_at	-1.972	163.245	SFG
EP300	202221_s_at	1.983	151.633	MTG, SFG
ZC3H11A	205787_x_at	1.963	147.34	EC, MTG
KLF9	203542_s_at	2.02	143.819	MTG
ZFR	201856_s_at	1.987	141.377	MTG, SFG
NFAT5	208003_s_at	1.976	138.656	PC, MTG, SFG
ZNF710	39891_at	1.989	134.643	PC, MTG
YY1	201901_s_at	1.965	133.824	MTG
ZNF609	212620_at	1.984	118.809	MTG, SFG
ZNF785	1554770_x_at	1.887	117.578	MTG, SFG
ZNF671	219849_at	-1.919	109.466	PC
ZNF562	219163_at	1.914	109.206	MTG, SFG
THRA	1316_at	1.969	105.009	PC, MTG, SFG
ZFP91	224631_at	1.96	103.026	MTG
KHSRP	204372_s_at	1.937	102.221	PC, MTG
ZBTB7A	213303_x_at	2.059	98.331	MTG
BUD31	205690_s_at	-1.876	94.581	PC, MTG
ZNF777	1553172_at	1.98	92.025	MTG
PHF3	215718_s_at	1.999	82.351	MTG, SFG
ZNF319	228460_at	1.992	80.796	MTG
BBX	232008_s_at	1.943	72.571	MTG
ILF3	208930_s_at	2.09	65.609	MTG
ZNF264	205917_at	1.9	60.619	MTG, SFG
TRIM27	212118_at	-1.877	56.333	PC, MTG
SOX5	207336_at	2.042	55.583	MTG
ZDHHC21	233216_at	1.988	55.417	MTG, SFG
BBX	223134_at	1.967	55.002	EC, MTG, SFG
IKZF4	226759_at	1.895	53.461	SFG
ZFC3H1	213065_at	-1.959	47.312	PC
ZBED5	218263_s_at	-1.81	42.383	PC
FOXK2	226224_at	-1.871	41.367	PC
ZNF75A	227670_at	-1.893	37.534	PC
LMO3	204424_s_at	-1.937	34.285	PC, MTG
ZNF410	202010_s_at	-1.885	33.943	PC
ZCCHC17	223107_s_at	-1.829	31.673	PC, MTG
ZNF266	214686_at	-1.931	29.573	PC
ZNF561	235200_at	2.017	28.886	MTG
GZF1	225884_s_at	-1.808	28.467	PC
ZNF451	215012_at	1.89	27.976	MTG
NDAD versus Affected				

TSC22D4	208104_s_at	1.806	46.219	EC, MTG, SFG
ZNF358	219379_x_at	1.837	45.177	EC, PC, MTG, SFG, VCX
TFE3	212457_at	1.881	38.376	MTG, SFG, VCX
NFIC	213298_at	1.902	32.403	EC, MTG, SFG, VCX
HEY2	222921_s_at	1.798	31.96	EC, MTG, SFG
WIZ	52005_at	1.83	31.511	MTG, SFG, VCX
HIF3A	219319_at	1.774	31.032	EC, MTG, SFG, VCX
NFIC	206929_s_at	1.824	30.317	EC, MTG, SFG, VCX
ZFYVE20	1553570_x_at	1.839	28.904	EC, MTG, SFG, VCX
ILF3	217804_s_at	1.938	27.087	VCX
MAZ	212064_x_at	1.921	27.083	EC, PC, SFG, VCX
NFIA	224970_at	1.801	25.591	EC, MTG, SFG, VCX
HDGF	200896_x_at	1.901	25.178	EC, MTG, SFG, VCX
NFIA	226806_s_at	1.783	24.151	EC, MTG, SFG, VCX
ZBTB4	227047_x_at	1.942	23.072	SFG, VCX
NR2F6	209262_s_at	1.86	22.529	MTG, VCX
TCF7L1	221016_s_at	1.847	21.478	EC, MTG, SFG, VCX
SIX5	229009_at	1.736	20.552	EC, SFG, VCX
ZDHHC21	229240_at	-1.701	19.501	PC, MTG, VCX
BAZ1B	213336_at	1.877	18.534	EC
USF2	202152_x_at	1.863	16.867	EC, SFG, VCX
NR2F1	209506_s_at	1.81	16.799	MTG, VCX
TFEB	50221_at	1.773	16.693	PC, SFG
ZNF385A	226111_s_at	1.81	13.899	EC, PC
Control versus NDAD				
KDM5A	215698_at	1.976	191.759	MTG
ZNF780B	244818_at	1.877	188.161	PC, VCX
TFAM	203176_s_at	2.118	183.427	MTG
ZBED1	203043_at	-2.018	173.732	VCX
ZFR	33148_at	2.12	162.078	MTG
SNAPC1	205443_at	2.048	152.918	MTG
IKZF4	226759_at	1.871	151.77	MTG
ZNF638	1554249_a_at	2.04	144.44	MTG
ZNF589	1569108_a_at	1.885	140.579	MTG
ZNF770	238687_x_at	2.066	127.226	MTG
BCLAF1	214499_s_at	2.02	124.584	MTG
MLL	212079_s_at	2.002	106.517	MTG
MEF2A	208328_s_at	2.002	104.843	MTG
ZNF800	227101_at	2.003	102.538	MTG
BCLAF1	201083_s_at	2.04	88.684	MTG
ZNF264	205917_at	1.874	83.076	MTG
ZFP14	232911_at	1.87	80.967	MTG
ZFAND5	217741_s_at	1.996	75.424	MTG
MEF2D	225641_at	2.004	74.555	EC, PC, MTG, VCX
THRA	204100_at	-1.884	72.805	VCX
KDM5B	201548_s_at	2.053	72.147	MTG
ZMYM2	210282_at	2.087	68.367	MTG

ATF4	200779_at	-1.902	67.54	VCX
RUNX1T1	205529_s_at	1.976	64.892	MTG
KLF9	203542_s_at	1.938	53.226	MTG
NCOA1	210249_s_at	-1.967	50.224	VCX
ZBTB38	1558733_at	1.966	50.108	MTG
RUNX1T1	205528_s_at	2.025	48.329	MTG
ZMYM3	1554171_at	1.967	45.918	EC, PC, MTG, SFG, VCX
ILF3	217804_s_at	-1.943	45.026	MTG
LHX2	211219_s_at	-1.873	42.066	EC
ZNF587	231820_x_at	1.902	41.494	MTG
ZCCHC2	219062_s_at	2.048	40.86	PC, MTG, VCX
ZDHHC21	233216_at	1.945	40.791	MTG, SFG
ZNF319	228460_at	2.046	40.054	MTG
NCOA1	209107_x_at	-1.904	39.312	VCX
EP300	202221_s_at	1.895	38.002	MTG
NFIA	224976_at	-1.903	36.78	PC, VCX
BCL11A	219498_s_at	1.993	36.541	PC, MTG
ZNF777	1553172_at	2.113	36.529	MTG
LZTS1	47550_at	2.054	34.476	MTG
ZNF320	229614_at	1.941	33.659	EC, MTG
ZDHHC21	243835_at	2.026	32.69	MTG
ZFYVE21	224445_s_at	-1.97	31.31	PC
BCL11A	222891_s_at	1.972	30.924	MTG
TSC22D2	204094_s_at	2.039	25.448	MTG
GATAD2B	225393_at	2.108	25.185	MTG
SALL2	213283_s_at	-1.97	24.997	EC
ZBTB16	205883_at	-1.962	23.907	VCX
ZFAND3	218020_s_at	-2.098	22.06	PC
THRA	35846_at	-1.986	21.925	EC, PC, VCX
RBPJ	211974_x_at	-2.009	20.133	EC, PC, MTG, VCX
ZC3HAV1L	228280_at	1.941	13.91	MTG
CTBP1	213980_s_at	-2.297	8.956	EC, PC

MR master regulator, NES normalized enrichment score, HIP hippocampus, AD Alzheimer’s disease, NDAD non-demented Alzheimer’s disease
