# Supplementary material for: Assembly and Interrogation of Alzheimer’s Disease Genetic Networks Reveal Novel Regulators of Progression
Source: PLoS One. 2015 Mar 17;10(3):e0120352. doi: 10.1371/journal.pone.0120352 (PMC4363671; doi:10.1371/journal.pone.0120352)
Supplement: S3 Table — (PDF) [file pone.0120352.s009.pdf]

Gene Name	Probe Number	NES	Odds Ratio	Additional Regions
Control versus Affected				
CITED1	207144_s_at	-1.806	69.084	MTG, SFG
ZXDC	234991_at	1.936	46.381	MTG
MITF	226066_at	1.994	44.162	MTG
TCFL5	235694_at	1.88	43.164	MTG
VEZF1	202173_s_at	1.937	39.933	MTG
ZFHX3	226137_at	1.908	33.487	MTG, SFG
TSC22D2	204094_s_at	1.907	30.891	HIP, MTG
LEF1	221558_s_at	1.917	29.768	SFG
ZCCHC24	212423_at	1.847	26.856	MTG, SFG
BBX	213015_at	1.918	25.219	HIP, MTG, SFG
CREBBP	202160_at	1.92	22.095	MTG, SFG
HES6	226446_at	1.891	21.327	MTG
RORA	235567_at	1.882	20.527	HIP, MTG
ZBTB20	222357_at	1.891	17.512	MTG, SFG
PHF21B	1562309_s_at	2.212	17.432	SFG
TRPS1	222651_s_at	1.91	16.189	MTG
ETS1	224833_at	1.894	14.628	MTG
NDAD versus Affected				
SOX9	202935_s_at	1.829	112.6	MTG, SFG, VCX
ELF1	212420_at	1.698	97.189	MTG, SFG
BAZ1B	213336_at	1.757	96.964	HIP
TSC22D4	208104_s_at	1.907	85.418	HIP, MTG, SFG
HES1	203394_s_at	1.929	80.666	HIP, MTG, SFG, VCX
SOX10	209842_at	1.826	77.052	PC, MTG, SFG, VCX
USF2	214879_x_at	1.802	73.327	HIP, SFG, VCX
ZBTB24	205340_at	-1.732	70.819	MTG
ZDHHC21	235068_at	-1.776	70.288	VCX
ZNF358	219379_x_at	1.775	69.593	HIP, PC, MTG, SFG, VCX
NFIA	226806_s_at	1.806	63.533	HIP, MTG, SFG, VCX
USF2	202152_x_at	1.875	63.317	HIP, SFG, VCX
GLI2	228537_at	1.744	62.957	SFG
LMO4	227155_at	-1.723	60.375	SFG
ZNF529	231940_at	-1.736	59.04	MTG, VCX
TFE3	206649_s_at	1.843	57.891	MTG, SFG
NFIA	224970_at	1.793	48.378	HIP, MTG, SFG, VCX
ATOH8	228890_at	1.716	47.569	MTG, SFG
KLF15	231015_at	1.675	45.079	MTG, SFG
ZFYVE20	1553569_at	1.785	44.878	SFG, VCX
ZNF623	206188_at	-1.734	44.422	MTG, VCX
HDGF	216484_x_at	1.764	43.532	HIP, SFG, VCX
ZFHX3	242738_s_at	1.727	43.425	SFG
ZFYVE20	1553570_x_at	1.831	43.339	HIP, MTG, SFG, VCX
ZCCHC24	212423_at	1.788	42.234	MTG, SFG

RXRA	202449_s_at	1.698	41.54	MTG, SFG, VCX
FOXO4	205451_at	1.664	41.137	PC
LMO7	202674_s_at	-1.734	40.757	MTG, SFG, VCX
MITF	207233_s_at	1.928	40.364	MTG, SFG, VCX
MECOM	221884_at	1.748	39.747	MTG, SFG, VCX
NKX2-2	206915_at	1.775	39.689	MTG, SFG
HIF3A	219319_at	1.709	39.466	HIP, MTG, SFG, VCX
MTF2	203347_s_at	-1.798	38.725	MTG
ZC3H14	213063_at	-1.705	38.678	PC
HIF3A	1556069_s_at	1.717	38.408	MTG, SFG
ZNF711	228988_at	-1.704	36.259	MTG, SFG, VCX
ZCCHC24	212419_at	1.733	35.595	MTG, SFG, VCX
SIX5	229009_at	1.713	35.008	HIP, SFG, VCX
ARID5A	213138_at	1.683	34.589	PC
HIF3A	232669_at	1.751	34.148	MTG, SFG
ZMYM6	227594_at	-1.675	33.508	HIP
BLZF1	203840_at	-1.73	33.144	MTG, VCX
HEY2	222921_s_at	1.731	31.673	HIP, MTG, SFG
ZFP36L1	211962_s_at	1.718	30.895	MTG, SFG, VCX
SOX15	217040_x_at	1.627	29.994	PC
ZNF382	1557260_a_at	-1.697	27.925	MTG, SFG, VCX
PPARA	244689_at	1.827	24.531	MTG
SATB2	213435_at	-1.723	22.147	PC, SFG
GATA2	209710_at	1.683	21.934	MTG, SFG
TCF7L2	212762_s_at	1.796	20.504	PC, MTG, SFG, VCX
HDGF	200896_x_at	1.793	19.315	HIP, MTG, SFG, VCX
UBTF	225982_at	1.675	17.725	MTG
ZBTB20	222357_at	1.765	14.97	MTG, SFG
TBX2	40560_at	1.764	14.411	SFG
CUX1	202367_at	1.913	13.41	SFG
ZBTB20	205383_s_at	1.812	10.925	MTG, SFG, VCX
Control versus NDAD				
HMBOX1	219269_at	-1.823	73.231	HIP
MEF2A	214684_at	1.764	71.763	HIP, MTG
ZMYM2	202778_s_at	1.769	57.292	VCX
ZNF720	238510_at	1.711	43.792	PC, MTG
ZNF281	228785_at	1.66	42.511	MTG
ZMYM3	1554171_at	1.777	37.544	HIP, PC, MTG, SFG, VCX
ZFHX4	219779_at	-1.902	33.871	PC, VCX
LHX2	211219_s_at	-1.82	33.328	HIP
BUD31	215815_at	-1.602	32.385	MTG
SOX2	213721_at	-1.839	23.11	HIP, PC
RBPJ	211974_x_at	-1.808	22.083	HIP, PC, MTG, VCX
CTBP1	213980_s_at	1.777	21.756	HIP, PC
MEF2D	225641_at	1.986	13.687	HIP, PC, MTG, VCX

MR master regulator, NES normalized enrichment score, EC entorhinal cortex, AD Alzheimer's disease, NDAD non-demented Alzheimer's disease
