# Supplementary material for: Assembly and Interrogation of Alzheimer’s Disease Genetic Networks Reveal Novel Regulators of Progression
Source: PLoS One. 2015 Mar 17;10(3):e0120352. doi: 10.1371/journal.pone.0120352 (PMC4363671; doi:10.1371/journal.pone.0120352)
Supplement: S4 Table — (PDF) [file pone.0120352.s010.pdf]

Gene Name	Probe Number	NES	Odds Ratio	Additional Regions
Control versus Affected				
ZNF671	219849_at	-1.839	Inf	HIP
ZBTB7A	219186_at	1.859	103.182	MTG
ZNF10	229848_at	-1.887	99.875	SFG
ZNF512	225050_at	-1.891	93.853	HIP
HOPX	211597_s_at	-1.831	92.986	MTG
TCF7	205255_x_at	1.883	92.086	MTG, SFG
MNT	204206_at	1.855	91.746	SFG
ZDHHC6	218249_at	-1.841	88.808	SFG
ZNF713	1560201_at	1.796	84.233	HIP
ZNF710	39891_at	1.92	81.482	HIP, MTG
BRD8	242265_at	1.841	79.7	HIP, MTG, SFG
ZXDC	235448_at	1.816	74.596	MTG, SFG
ATF7IP	231825_x_at	1.828	73.263	MTG, SFG
RAX2	1552311_a_at	1.832	71.647	SFG
MXD4	212346_s_at	1.837	71.505	MTG, SFG
PHTF1	205702_at	-1.81	69.695	HIP, MTG
JUND	203752_s_at	1.897	68.871	MTG
PRDM4	218329_at	-1.83	68.853	MTG
SNAPC5	213203_at	-1.778	65.431	SFG
HOPX	1566140_at	1.875	65.271	HIP, MTG
BUD31	205690_s_at	-1.833	64.129	HIP, MTG
LRPPRC	211615_s_at	-1.811	59.276	MTG
ZBED5	218263_s_at	-1.813	58.481	HIP
ZFP90	226124_at	-1.865	57.384	HIP
RNF4	212696_s_at	-1.86	55.105	HIP
TFCP2	209338_at	-1.83	52.794	SFG
ZNF160	214715_x_at	1.777	51.311	MTG, SFG
ZEB2	233031_at	1.857	50.38	HIP, SFG
ASH2L	209517_s_at	-1.818	49.381	HIP, MTG
ZNF263	203707_at	-1.833	49.314	HIP
HNRNPAB	201277_s_at	-1.826	48.648	HIP
TULP4	218184_at	-1.781	43.284	HIP
RFX1	226786_at	1.759	43.124	HIP
SOLH	204275_at	1.905	41.642	SFG
ZFAND2A	226650_at	-1.784	41.45	HIP, MTG
ZNF827	243618_s_at	1.827	39.378	HIP
ZBTB47	226484_at	1.85	39.017	MTG, SFG, VCX
KHSRP	212303_x_at	1.797	38.725	HIP
EDF1	209059_s_at	-1.798	37.29	HIP
PPARD	37152_at	1.862	36.103	MTG, SFG
ZNF280D	239107_at	1.761	34.424	HIP, MTG, SFG
GZF1	225884_s_at	-1.795	33.957	HIP
ZNF205	206416_at	1.836	33.807	SFG
MEF2C	209200_at	-1.739	31.43	MTG

NDAD versus Affected				
ZRANB2	223716_s_at	-1.705	58.295	HIP
ZDHHC21	229240_at	-1.743	45.741	HIP, MTG, VCX
MBTPS2	226760_at	-1.751	43.208	VCX
MAZ	212064_x_at	1.855	41.517	HIP, EC, SFG, VCX
SOX10	209842_at	1.72	39.629	EC, MTG, SFG, VCX
PEG3	209242_at	-1.655	38.023	EC
ZDHHC17	212982_at	-1.746	35.239	EC
ZMYM4	202051_s_at	-1.688	34.822	EC
MAFF	205193_at	1.813	34.791	MTG, SFG
FOXO4	205451_at	1.742	32.907	EC
TFEB	50221_at	1.826	32.845	HIP, SFG
ARID5A	213138_at	1.897	28.862	EC
ZNF444	218707_at	1.739	28.719	EC
YEATS4	218911_at	-1.8	28.009	VCX
ZDHHC13	219296_at	-1.76	27.656	EC, MTG
TCF7L2	212759_s_at	1.824	26.033	EC, SFG, VCX
ZC3H14	213063_at	-1.75	25.904	EC
ZNF358	219379_x_at	1.741	25.316	HIP, EC, MTG, SFG, VCX
ZNF223	207128_s_at	-1.869	21.619	SFG, VCX
SOX15	217040_x_at	1.798	20.519	EC
LYL1	210044_s_at	1.665	17.701	SFG
Control versus NDAD				
ZNF254	1559449_a_at	2.026	82.953	MTG
ZNF814	242564_at	2.005	34.404	MTG, SFG
ZMYM3	1554171_at	1.939	32.916	HIP, EC, MTG, SFG, VCX
NR2E1	207443_at	-2.056	31.985	HIP
EMX2	221950_at	-2.016	25.57	HIP
PPARA	223437_at	-2.053	20.895	HIP
MEF2D	225641_at	2.047	18.83	HIP, EC, MTG, VCX
ZMYND11	202136_at	-2.062	17.919	VCX
NFIA	224976_at	-2.029	15.905	HIP, VCX
THRA	35846_at	-2.155	7.358	HIP, EC, VCX

MR master regulator, NES normalized enrichment score, PC posterior cingulate, AD Alzheimer’s disease, NDAD non-demented Alzheimer’s disease
