# Supplementary material for: Assembly and Interrogation of Alzheimer’s Disease Genetic Networks Reveal Novel Regulators of Progression
Source: PLoS One. 2015 Mar 17;10(3):e0120352. doi: 10.1371/journal.pone.0120352 (PMC4363671; doi:10.1371/journal.pone.0120352)
Supplement: S5 Table — (PDF) [file pone.0120352.s011.pdf]

Gene Name	Probe Number	NES	Odds Ratio	Additional Regions
Control versus Affected				
ZNF319	228460_at	1.992	305.415	HIP
MSRB2	219451_at	1.987	209.681	HIP
ASCL1	209988_s_at	1.859	208.326	SFG
ZNF665	220760_x_at	1.88	186.169	PC
BBX	223134_at	1.941	166.202	HIP, EC, SFG
ZNF462	244007_at	1.921	159.338	HIP, SFG
ZNF652	205594_at	1.948	146.591	HIP, SFG
ZFAND6	239757_at	1.937	143.279	SFG
NPAS3	220316_at	1.883	140.892	EC, SFG
JUND	203752_s_at	1.904	135.507	PC
SOX2	228038_at	1.798	133.124	SFG
SOX5	207336_at	1.896	121.616	HIP
ZNF462	226575_at	1.97	120.368	SFG
ZNF561	235200_at	1.984	116.711	HIP
RFX4	223673_at	1.813	114.618	SFG
FOXO1	202724_s_at	1.831	112.083	SFG
BBX	213015_at	1.908	107.72	HIP, EC, SFG
TCFL5	235694_at	1.946	103.897	EC
PPARA	226978_at	1.864	98.802	SFG
TAL1	206283_s_at	1.838	92.281	SFG
TCF3	209153_s_at	1.884	87.66	PC, SFG
CREBBP	202160_at	1.916	86.53	EC, SFG
ZEB2	203603_s_at	1.897	85.687	EC
ZBTB47	226484_at	1.881	82.799	PC, SFG, VCX
EGR1	227404_s_at	-1.863	79.808	SFG
SOX9	202936_s_at	1.835	78.652	SFG
NFIA	224975_at	1.86	77.804	EC, SFG
ZFHX3	226137_at	1.894	77.658	EC, SFG
HIF3A	1555318_at	1.849	77.587	SFG
MAFF	36711_at	1.864	75.969	SFG
BAZ1A	217985_s_at	1.869	75.498	SFG
ZNF776	242992_at	1.887	74.297	SFG
PPARD	37152_at	1.905	72.076	PC, SFG
ZNF641	235179_at	1.895	69.222	HIP
SP3	232529_at	1.892	68.759	SFG
ZNF584	228148_at	-1.872	68.616	SFG
ZFP36L1	211962_s_at	1.849	67.219	SFG
ZC3H11A	205787_x_at	1.941	66.512	HIP, EC
WIZ	52005_at	1.89	65.799	HIP, SFG
ILF3	208930_s_at	1.957	65.316	HIP
NFIB	209290_s_at	1.959	64.639	SFG
ASH2L	209517_s_at	-1.854	62.242	HIP, PC
ZNF562	219163_at	1.9	61.782	HIP, SFG
ZXDC	234991_at	1.943	61.088	EC

YY1	201901_s_at	1.831	60.69	HIP
TCF4	212386_at	1.955	60.363	HIP, SFG
LMO3	204424_s_at	-1.911	58.384	HIP, PC
KLF9	203542_s_at	1.975	58.34	HIP
HOPX	1566140_at	1.895	58.117	HIP, PC
ZFAND6	222186_at	1.981	57.995	SFG
ZNF785	1554770_x_at	1.946	57.067	HIP, SFG
CITED1	207144_s_at	-1.832	56.226	EC, SFG
HBP1	236645_at	1.914	56.017	HIP, SFG
CTNNB1	223679_at	1.944	55.877	SFG
KLF15	231015_at	1.806	55.721	SFG
ZNF37A	228711_at	1.904	54.435	HIP, SFG
PHF5A	225309_at	1.863	54.199	HIP
THRA	1316_at	1.956	53.482	HIP, PC, SFG
POU3F2	207084_at	1.895	53.121	HIP
ZCCHC17	223107_s_at	-1.918	52.767	HIP, PC
SOX6	227497_at	1.842	52.693	SFG
ZNF131	1557384_at	1.892	49.735	HIP, PC, SFG
BUD31	205690_s_at	-1.878	49.679	HIP, PC
ZBTB7A	213303_x_at	2.002	48.419	HIP
PRDM2	205277_at	-1.819	47.614	HIP, PC
VEZF1	202173_s_at	1.892	47.464	EC
ZBTB1	205092_x_at	1.889	47.115	HIP
TCF7L2	212762_s_at	1.899	46.674	HIP, SFG
ZFAND2A	226650_at	-1.868	46.452	HIP, PC
ZMYND8	230533_at	1.914	45.285	HIP
PHF21A	203278_s_at	1.896	45.184	SFG
EP300	202221_s_at	1.86	43.278	HIP, SFG
ZDHHC21	233216_at	1.949	42.971	HIP, SFG
ZNF573	217627_at	1.874	42.225	HIP, SFG
SP1	224754_at	1.894	40.479	SFG
ZFR	201856_s_at	1.885	40.318	HIP, SFG
RORA	235567_at	1.969	39.776	HIP, EC
SP4	236265_at	1.839	38.553	HIP, SFG
ZNF274	232436_at	1.975	37.551	SFG
UHRF1	225655_at	1.871	36.876	EC, SFG
ZNF777	1553172_at	1.88	35.398	HIP
ZNF395	221123_x_at	1.883	34.825	SFG
PCGF2	214239_x_at	1.921	27.979	SFG
ZSCAN29	226562_at	1.933	27.253	EC
ZNF131	221842_s_at	1.849	26.04	HIP
AHCTF1	214766_s_at	1.981	25.65	HIP
NDAD versus Affected				
PATZ1	209431_s_at	1.932	Inf	SFG
AEBP1	201792_at	2.02	283.066	SFG
ZBBX	220269_at	-1.872	221.851	SFG
ELF1	212418_at	1.913	218.506	SFG

FOXO1	202724_s_at	1.926	212.95	SFG
PAX6	205646_s_at	1.91	209.173	SFG, VCX
TBX3	219682_s_at	1.855	206.156	SFG
PPARA	223437_at	1.887	204.863	SFG
MECOM	221884_at	1.985	201.809	EC, SFG, VCX
RELA	201783_s_at	1.922	201.625	SFG, VCX
NFIA	224975_at	1.91	197.842	SFG
FOXO1	202723_s_at	1.949	195.18	EC, SFG, VCX
ZCCHC24	212419_at	1.94	178.25	EC, SFG, VCX
EMX2	221950_at	1.876	171.576	SFG
ZNF621	1558620_at	1.939	153.169	EC, SFG
ZNF672	218068_s_at	1.976	151.146	SFG, VCX
SOX2	213721_at	1.921	144.616	SFG, VCX
SP1	224754_at	1.98	137.637	EC, SFG
LEF1	221558_s_at	1.934	135.386	SFG
HMG20B	210719_s_at	1.952	131.372	SFG, VCX
NFATC1	211105_s_at	1.962	122.539	SFG, VCX
MSX1	205932_s_at	1.921	121.615	SFG, VCX
ZFP36L1	211962_s_at	1.93	119.971	EC, SFG, VCX
ZNF382	1557260_a_at	-1.887	116.783	EC, SFG, VCX
CSDA	201161_s_at	1.868	113.69	SFG
RUNX1	209360_s_at	1.905	111.214	SFG
IRF7	208436_s_at	1.932	106.361	SFG, VCX
EPAS1	200878_at	1.919	104.47	SFG, VCX
MXD4	210778_s_at	1.896	101.232	EC, SFG, VCX
ZCCHC24	212423_at	1.935	100.173	EC, SFG
NFIA	226806_s_at	1.859	98.435	HIP, EC, SFG, VCX
HMBOX1	219269_at	1.94	97.962	SFG
TSC22D4	208104_s_at	1.911	95.878	HIP, EC, SFG
HIF3A	219319_at	1.897	95.002	HIP, EC, SFG, VCX
SMAD6	207069_s_at	1.907	90.849	SFG
FOXD1	206307_s_at	1.929	85.313	SFG, VCX
ASCL1	209987_s_at	1.918	85.016	SFG, VCX
NOTCH1	218902_at	1.842	81.033	SFG, VCX
ZBED3	228402_at	1.913	79.758	EC, SFG, VCX
EBF1	227646_at	1.983	76.225	SFG
NFIA	224970_at	1.882	75.92	HIP, EC, SFG, VCX
FOXC1	1553613_s_at	1.91	73.059	EC, SFG, VCX
ZBTB20	205383_s_at	1.937	72.774	EC, SFG, VCX
ZBED1	203043_at	1.864	69.586	SFG
ZNF529	231940_at	-1.771	69.123	EC, VCX
SOX17	230943_at	1.96	67.897	SFG
TCF7L1	221016_s_at	1.924	62.841	HIP, EC, SFG, VCX
CEBPD	203973_s_at	1.795	60.429	SFG
MAFF	36711_at	1.901	59.109	SFG
BBX	223135_s_at	1.966	58.782	SFG
ELF1	212420_at	1.939	58.265	EC, SFG
HDGF	200896_x_at	1.878	58.212	HIP, EC, SFG, VCX

STAT5A	203010_at	1.959	58.203	SFG
ZBTB20	235308_at	1.928	56.918	EC, SFG, VCX
ZNF462	226575_at	1.888	56.797	SFG
ZNF703	222760_at	2.008	55.132	SFG
RBPJ	211974_x_at	1.866	55.087	EC, SFG, VCX
HES1	203394_s_at	1.875	54.19	HIP, EC, SFG, VCX
HEY2	222921_s_at	1.804	53.44	HIP, EC, SFG
ZC3HAV1	213051_at	1.832	52.415	SFG, VCX
ZNF358	219379_x_at	1.849	51.413	HIP, EC, PC, SFG, VCX
NOTCH3	203238_s_at	1.879	49.18	EC, SFG
SP110	223980_s_at	1.924	49.157	SFG
HIF3A	232669_at	1.847	48.069	EC, SFG
LMO7	202674_s_at	-1.867	47.1	EC, SFG, VCX
ATF7IP2	228381_at	-1.877	46.758	SFG, VCX
ZDHHC13	219296_at	-1.928	44.837	EC, PC
CBL	225234_at	2.016	41.432	HIP, SFG, VCX
MITF	207233_s_at	1.905	40.662	EC, SFG, VCX
MEF2D	225641_at	-1.849	38.761	VCX
ATF2	212984_at	-1.78	38.314	VCX
NR2F2	209121_x_at	1.99	35.767	SFG, VCX
NFIX	228278_at	1.892	32.795	SFG, VCX
ZCCHC7	226496_at	-1.797	30.628	EC, SFG, VCX
TFE3	212457_at	1.987	29.481	HIP, SFG, VCX
ZNF684	244398_x_at	-1.937	28.997	VCX
MTF2	203347_s_at	-1.773	27.892	EC
BLZF1	203840_at	-1.822	25.285	EC, VCX
PLAGL1	207943_x_at	-1.852	24.119	VCX
TFE3	206649_s_at	1.838	24.073	EC, SFG
ZNF320	229614_at	-1.85	23.344	HIP, VCX
MITF	226066_at	2.009	22.195	SFG
NR2F6	209262_s_at	1.923	22.126	HIP, VCX
ZNF267	219540_at	-1.852	20.68	SFG, VCX
EGR1	201694_s_at	-1.771	20.555	SFG
ZNF395	221123_x_at	1.925	17.726	EC, SFG
ZMYM5	235620_x_at	-1.848	17.296	SFG, VCX
NFIC	213298_at	2.046	15.724	HIP, EC, SFG, VCX
FOXO3	204131_s_at	2.14	11.685	VCX
Control versus NDAD				
TSC22D2	204094_s_at	1.872	194.371	HIP
ZNF770	238687_x_at	1.898	191.16	HIP
ZNF566	240239_at	1.851	140.734	HIP
ZC3H14	213064_at	1.779	133.973	HIP
ZNF682	242915_at	1.844	124.519	HIP
IKZF4	226759_at	1.891	110.64	HIP
ZNF664	1558816_at	1.799	109.237	HIP
ZNF720	238510_at	1.858	102.894	EC, PC
LZTS1	47550_at	1.842	98.926	HIP

ZNF320	229614_at	1.817	95.158	HIP, EC
EP300	202221_s_at	1.833	89.525	HIP
ZCCHC2	219062_s_at	1.838	86.319	HIP, PC, VCX
ZNF561	235200_at	1.872	76.191	HIP
ZNF236	219171_s_at	1.789	75.6	HIP
ZNF587	231820_x_at	1.828	74.793	HIP
KDM5B	201548_s_at	1.835	70.936	HIP
ZNF814	60794_f_at	2.015	70.137	SFG
MEF2D	225641_at	1.868	68.49	HIP, EC, PC, VCX
ZNF264	205917_at	1.851	61.802	HIP
ZBTB1	205092_x_at	1.825	60.014	HIP
ZNF638	1554249_a_at	1.872	57.537	HIP
BCLAF1	201083_s_at	1.716	57.479	HIP
MSRB2	219451_at	1.878	54.56	HIP
NPAS2	39549_at	1.845	53.732	HIP
ZNF319	228460_at	1.917	51.294	HIP
ZMYM3	1554171_at	1.907	50.273	HIP, EC, PC, SFG, VCX
ZFR	33148_at	1.928	49.33	HIP
BCLAF1	214499_s_at	1.865	46.48	HIP
MEF2A	214684_at	1.798	45.38	HIP, EC
CNOT7	233019_at	1.784	43.274	HIP
GLIS1	244128_x_at	1.865	40.918	HIP
ZFAND5	217741_s_at	2.022	39.358	HIP
ZC3HAV1L	228280_at	1.846	34.45	HIP
ZNF800	227101_at	1.856	33.66	HIP
LMO3	204424_s_at	-1.859	30.824	HIP
BCL11A	222891_s_at	1.943	27.029	HIP
BUD31	215815_at	-1.726	26.425	EC
SNAPC1	205443_at	1.925	24.861	HIP
BCL11A	219498_s_at	1.865	21.749	HIP, PC
NFAT5	208003_s_at	1.857	21.411	HIP
RBPJ	211974_x_at	-1.864	19.69	HIP, EC, PC, VCX
ZBTB38	1558733_at	1.984	17.193	HIP

MR master regulator, NES normalized enrichment score, MTG middle temporal gyrus, AD Alzheimer's disease, NDAD non-demented Alzheimer's disease
