# Supplementary material for: Assembly and Interrogation of Alzheimer’s Disease Genetic Networks Reveal Novel Regulators of Progression
Source: PLoS One. 2015 Mar 17;10(3):e0120352. doi: 10.1371/journal.pone.0120352 (PMC4363671; doi:10.1371/journal.pone.0120352)
Supplement: S6 Table — (PDF) [file pone.0120352.s012.pdf]

Gene Name	Probe Number	NES	Odds Ratio	Additional Regions
Control versus Affected				
HIF3A	1555318_at	2.001	227.508	MTG
PHF3	215718_s_at	2.18	182.445	HIP, MTG
PPARA	226978_at	1.993	165.462	MTG
MYT1	210341_at	1.982	126.465	MTG
FOXO1	202724_s_at	1.971	120.586	MTG
ZXDC	235448_at	2.047	105.537	PC, MTG
TAL1	206283_s_at	2.022	97.853	MTG
AEBP1	201792_at	1.955	97.573	MTG
SOX2	228038_at	1.972	96.957	MTG
ZFP36L1	211962_s_at	2.053	94.97	MTG
RFX4	223673_at	1.936	88.472	MTG
MECOM	221884_at	1.997	87.36	MTG
ZHX2	1557706_at	1.868	86.45	PC, MTG
ZHX2	203556_at	1.924	86.158	MTG
NOTCH1	218902_at	1.916	83.581	MTG
CEBPD	203973_s_at	2.004	80.32	MTG
ZNF584	228148_at	-2.05	78.1	MTG
NOTCH2	202443_x_at	1.908	76.188	MTG
MXD4	212346_s_at	1.903	74.945	PC, MTG
SOX9	202936_s_at	1.93	74.51	MTG
ZFAND6	222186_at	2.068	74.068	MTG
BCL6	203140_at	2.048	71.221	EC, MTG
TEAD2	226408_at	1.842	68.264	MTG
KLF2	219371_s_at	1.891	66.605	MTG
NFIA	226806_s_at	1.975	65.755	MTG
ZNF785	1554770_x_at	2.112	64.13	HIP, MTG
MAFF	36711_at	1.974	63.979	MTG
GATAD2B	238076_at	2.104	63.712	MTG
BCL6	228758_at	2.08	60.722	MTG
NOTCH2	212377_s_at	1.914	60.716	MTG
NPAS3	220316_at	1.949	59.156	EC, MTG
ZNF618	238444_at	2.074	59.116	MTG
ZNF462	226575_at	1.913	58.832	MTG
HBP1	236645_at	2.162	58.665	HIP, MTG
ZNF160	214715_x_at	2.029	57.595	PC, MTG
ZNF652	205594_at	1.955	56.781	HIP, MTG
ZNF397OS	1557104_at	2.031	56.679	PC, MTG
TCF3	209153_s_at	1.913	56.434	PC, MTG
VEZF1	202171_at	2.001	56.273	MTG
ZDHHC6	218249_at	-1.838	55.645	PC
NFIA	224975_at	1.954	53.886	EC, MTG
ZNF785	1554769_at	2.123	52.767	HIP, MTG
BAZ1A	217985_s_at	2.017	52.591	MTG
ZNF274	232436_at	1.97	51.482	MTG

ZFAND6	239757_at	2.014	50.626	MTG
NFIA	224970_at	1.909	49.064	MTG
ZMIZ2	54970_at	1.994	47.177	MTG
ZNF462	244007_at	1.918	46.711	HIP, MTG
PHF11	242060_x_at	1.933	45.92	HIP, PC, MTG
ZNF449	228968_at	-2.006	45.811	PC, VCX
ZNF444	50376_at	1.932	45.463	PC, MTG, VCX
ZNF207	239937_at	1.984	45.369	PC
BRD8	242265_at	1.918	44.616	HIP, PC, MTG
ZBTB47	226484_at	1.924	44.403	PC, MTG, VCX
LHX2	206140_at	1.927	43.241	MTG
ZNF230	205791_x_at	-1.869	42.483	PC
ASCL1	209988_s_at	1.941	41.913	MTG
TFEB	50221_at	1.936	41.859	HIP, PC, MTG, VCX
ZNF562	219163_at	2.137	41.716	HIP, MTG
ZC3H11A	1565867_a_at	2.048	40.095	HIP
BBX	223134_at	2.05	39.371	HIP, EC, MTG
LEF1	221558_s_at	1.962	38.647	EC
JARID2	203297_s_at	2.007	38.282	EC
ZBTB20	235308_at	1.957	35.951	MTG
PPARD	37152_at	1.892	35.168	PC, MTG
ZC3H7B	206169_x_at	2.047	35.092	PC
WIZ	52005_at	1.907	33.905	HIP, MTG
ZNF609	212620_at	2.027	33.201	HIP, MTG
RFX5	202963_at	-1.868	33.183	PC
ZNF37A	228711_at	2.054	31.895	HIP, MTG
ZNF280D	239107_at	1.898	31.759	HIP, PC, MTG
HEY2	222921_s_at	1.931	31.006	MTG
ZNF611	208137_x_at	1.939	30.832	PC
SOX9	202935_s_at	1.933	30.829	MTG
PHTF2	217097_s_at	2.143	30.341	HIP
ZNF205	206416_at	1.941	30.276	PC
MTF1	205322_s_at	2.011	29.11	HIP, MTG
ZEB2	233031_at	1.916	28.954	HIP, PC
CEBPB	212501_at	2.031	28.465	EC, MTG
ZNF10	229848_at	-1.778	28.156	PC
THRA	1316_at	2.014	27.797	HIP, PC, MTG
CITED1	207144_s_at	-1.857	27.242	EC, MTG
NR2F1	209506_s_at	2.003	26.92	HIP
EGR1	227404_s_at	-1.818	26.862	MTG
ZBTB20	222357_at	1.927	25.965	EC, MTG
PHF21A	203278_s_at	2.067	25.944	MTG
TFE3	212457_at	2.07	25.876	MTG
NR2F2	209119_x_at	1.781	25.103	HIP, PC, MTG
ZNF395	221123_x_at	2.082	23.451	MTG
ZMYM5	206652_at	2.051	21.935	HIP, MTG
ZNF573	217627_at	2.072	21.79	HIP, MTG
SP110	223980_s_at	1.934	20.941	MTG

ZNF207	231848_x_at	2.142	19.031	HIP, MTG
HIC2	212964_at	1.943	18.439	MTG
PCGF2	214239_x_at	2.251	18.044	MTG
ZDHHC21	233216_at	2.142	16.892	HIP, MTG
NEUROD6	220045_at	-1.935	13.344	MTG
NDAD versus Affected				
PPARA	226978_at	2.013	804.803	VCX
PATZ1	209431_s_at	1.963	243.482	MTG
TSC22D1	235315_at	-2.047	235.091	MTG
FOXO1	202724_s_at	1.959	185.435	MTG
FOXO1	202723_s_at	1.978	177.648	EC, MTG, VCX
TCF7L1	221016_s_at	1.985	168.925	HIP, EC, MTG, VCX
AEBP1	201792_at	2.02	166.073	MTG
IRF7	208436_s_at	1.96	163.109	MTG, VCX
SP110	208012_x_at	1.924	162.505	MTG
ZNF621	1558620_at	1.955	162.494	EC, MTG
NPAS3	229281_at	1.946	153.708	MTG
PPARA	223437_at	2.004	150.525	MTG
PAX6	205646_s_at	1.979	130.274	MTG, VCX
MECOM	221884_at	1.999	129.775	EC, MTG, VCX
NPAS3	230412_at	1.977	128.236	MTG
GATAD2A	218131_s_at	1.998	125.283	MTG
RELA	201783_s_at	1.944	124.18	MTG, VCX
NFIA	224970_at	1.899	123.437	HIP, EC, MTG, VCX
ZFP36L1	211962_s_at	1.932	119.491	EC, MTG, VCX
EMX2	221950_at	1.952	114.578	MTG
NFIA	224975_at	1.953	112.779	MTG
SOX2	213721_at	1.962	111.367	MTG, VCX
ZC3HAV1	225634_at	2.018	109.806	MTG
LASS2	222212_s_at	1.989	107.52	VCX
CSDA	201161_s_at	1.94	101.219	MTG
ZBBX	220269_at	-1.96	100.227	MTG
GATAD2A	234294_x_at	2.036	98.263	MTG
LEF1	221558_s_at	1.975	97.435	MTG
NKX2-2	206915_at	1.888	95.563	EC, MTG
ELF1	212420_at	1.864	91.771	EC, MTG
NOTCH2	212377_s_at	1.997	90.292	MTG
ZNF672	218068_s_at	2.033	90.242	MTG, VCX
BCL6	228758_at	2.033	88.677	MTG, VCX
HIF3A	1555318_at	1.932	87.675	MTG
ZCCHC24	212419_at	1.916	86.228	EC, MTG, VCX
NOTCH3	203238_s_at	1.852	84.899	EC, MTG
HIF3A	219319_at	1.868	84.502	HIP, EC, MTG, VCX
HMBOX1	219269_at	1.931	84.194	MTG
ZHX2	203556_at	1.905	83.359	EC, VCX
ZIC1	206373_at	1.932	81.385	MTG
ZCCHC24	212423_at	1.983	79.643	EC, MTG

ZFP36L2	201368_at	1.987	79.608	MTG
NFIA	226806_s_at	1.883	79.145	HIP, EC, MTG, VCX
LMO7	202674_s_at	-1.839	76.737	EC, MTG, VCX
ZNF358	219379_x_at	1.894	75.099	HIP, EC, PC, MTG, VCX
RXRA	202449_s_at	1.909	75.098	EC, MTG, VCX
HMG20B	210719_s_at	1.974	74.671	MTG, VCX
ELF1	212418_at	1.963	74.653	MTG
ZBTB20	235308_at	1.973	73.432	EC, MTG, VCX
NOTCH1	218902_at	1.828	73.205	MTG, VCX
MAFF	36711_at	1.963	73.011	MTG
TBX2	40560_at	1.828	72.309	EC
ZFHX3	242738_s_at	1.846	71.796	EC
ATOH8	228890_at	1.772	71.751	EC, MTG
EBF1	227646_at	1.93	70.484	MTG
ZC3HAV1	213051_at	1.991	66.876	MTG, VCX
NFIB	209290_s_at	2.052	66.725	MTG
FOXC1	1553613_s_at	1.88	66.045	EC, MTG, VCX
SP1	224754_at	2.02	65.052	EC, MTG
BAZ2A	201353_s_at	1.999	63.838	VCX
ZNF382	1557260_a_at	-1.902	63.425	EC, MTG, VCX
ZNF382	1561687_a_at	-1.973	63.315	PC, VCX
HEY2	219743_at	1.925	62.417	MTG
RUNX1	209360_s_at	2.165	60.856	MTG
BCL6	203140_at	2.037	60.225	MTG
TBX3	219682_s_at	1.942	59.604	MTG
SP110	223980_s_at	2.017	58.51	MTG
EBF1	229487_at	1.861	57.179	EC
VEZF1	202173_s_at	1.988	53.126	MTG
EPAS1	200878_at	1.945	52.958	MTG, VCX
SOX9	202935_s_at	1.89	51.992	EC, MTG, VCX
STAT3	208992_s_at	2.041	51.488	EC, MTG, VCX
CBL	225234_at	2.037	51.083	HIP, MTG, VCX
ZFHX3	226137_at	1.913	50.815	EC, MTG
BBX	223135_s_at	2.024	48.518	MTG
HES1	203394_s_at	1.923	48.204	HIP, EC, MTG, VCX
ZFHX4	219779_at	1.978	47.209	MTG, VCX
MXD4	210778_s_at	1.834	46.994	EC, MTG, VCX
TFEB	50221_at	1.834	46.441	HIP, PC
ASCL1	209987_s_at	1.942	46.257	MTG, VCX
TFB1M	219169_s_at	-1.941	46.127	MTG
ZBTB20	205383_s_at	1.946	46.021	EC, MTG, VCX
GLI2	228537_at	1.911	45.614	EC
FOXD1	206307_s_at	1.941	45.564	MTG, VCX
CSDA	201160_s_at	1.962	45.159	MTG
ZBED1	203043_at	1.976	45.076	MTG
CEBPD	203973_s_at	1.868	43.645	MTG
ZC3H7B	205877_s_at	1.936	43.568	VCX
FOXC1	213260_at	1.868	42.614	MTG

MITF	207233_s_at	2.012	42.557	EC, MTG, VCX
HEY2	222921_s_at	1.911	40.8	HIP, EC, MTG
TBX2	213417_at	1.906	40.491	MTG
SOX10	209842_at	1.835	39.281	EC, PC, MTG, VCX
ZFAND6	239757_at	2.095	39.004	MTG
HEYL	226828_s_at	1.82	38.73	EC, MTG
NFIC	206929_s_at	1.934	38.609	HIP, EC, MTG, VCX
HDGF	200896_x_at	1.919	38.233	HIP, EC, MTG, VCX
TCF7L2	236094_at	1.894	38.225	MTG, VCX
NR2F2	209121_x_at	1.969	37.972	MTG, VCX
TFE3	212457_at	1.993	37.62	HIP, MTG, VCX
ZNF449	228968_at	-1.976	37.531	VCX
ZBTB4	227047_x_at	2.022	36.201	HIP, VCX
SOX17	230943_at	1.898	35.899	MTG
ZNF444	50376_at	1.83	35.75	MTG, VCX
HIF3A	232669_at	1.859	35.351	EC, MTG
WIZ	52005_at	1.871	35.126	HIP, MTG, VCX
ZNF703	222760_at	1.893	33.554	MTG
MXI1	202364_at	2.015	32.919	MTG, VCX
RBPJ	211974_x_at	1.98	32.607	EC, MTG, VCX
MTF1	205322_s_at	1.939	32.076	MTG, VCX
RBCK1	207713_s_at	1.943	32.028	HIP, VCX
ZFYVE20	1553569_at	1.913	31.989	EC, VCX
ZNF711	228988_at	-1.745	31.857	EC, MTG, VCX
STAT5A	203010_at	1.936	31.461	MTG
ZFYVE20	1553570_x_at	1.981	29.939	HIP, EC, MTG, VCX
ZNF223	207128_s_at	-1.985	24.98	PC, VCX
TFE3	206649_s_at	1.972	24.917	EC, MTG
USF2	202152_x_at	1.921	23.292	HIP, EC, VCX
NFIC	213298_at	2.137	22.136	HIP, EC, MTG, VCX
EGR1	201694_s_at	-1.832	21.551	MTG
TCF7L2	212762_s_at	2.033	20.514	EC, PC, MTG, VCX
ZCCHC7	226496_at	-1.936	20.488	EC, MTG, VCX
LMO2	204249_s_at	1.963	16.862	MTG
MAZ	212064_x_at	1.91	16.606	HIP, EC, PC, VCX
ZNF267	219540_at	-1.896	16.593	MTG, VCX
EBF1	232204_at	1.881	16.542	EC, MTG
ZNF395	218149_s_at	1.986	15.302	EC
ZNF395	223216_x_at	1.969	14.482	EC, MTG
Control versus NDAD				
ATF6B	1554487_a_at	2.059	57.217	PC
ZNF814	242564_at	2.08	46.199	PC, MTG
ZNF814	60794_f_at	2.186	18.153	MTG
ZMYM3	1554171_at	2.139	16.525	HIP, EC, PC, MTG, VCX
ZDHHC21	233216_at	2.052	10.659	HIP, MTG

MR master regulator, NES normalized enrichment score, SFG superior frontal gyrus, AD Alzheimer’s disease, NDAD non-demented Alzheimer’s disease
