# Supplementary material for: Assembly and Interrogation of Alzheimer’s Disease Genetic Networks Reveal Novel Regulators of Progression
Source: PLoS One. 2015 Mar 17;10(3):e0120352. doi: 10.1371/journal.pone.0120352 (PMC4363671; doi:10.1371/journal.pone.0120352)
Supplement: S7 Table — (PDF) [file pone.0120352.s013.pdf]

| Gene Name | Probe Number | NES | Odds Ratio | Additional Regions |
| --- | --- | --- | --- | --- |
| Control versus Affected | | | | |
| ZC3H13 | 227536_at | -1.762 | 48.506 | PC |
| ZNF449 | 228968_at | -1.747 | 30.925 | PC, SFG |
| ZNF226 | 219603_s_at | -1.775 | 30.432 | PC |
| PHF20L1 | 226942_at | -1.753 | 29.047 | PC |
| ZBTB47 | 226484_at | 1.75 | 28.159 | PC, MTG, SFG |
| ZNF419 | 58367_s_at | -1.68 | 16.248 | PC |
| NDAD versus Affected | | | | |
| PPARA | 226978_at | 1.874 | 96.56 | SFG |
| HDGF | 200896_x_at | 1.941 | 81.516 | HIP, EC, MTG, SFG |
| ZNF672 | 218068_s_at | 2.053 | 77.909 | MTG, SFG |
| FOXO1 | 202723_s_at | 1.91 | 63.711 | EC, MTG, SFG |
| MSX1 | 205932_s_at | 1.813 | 63.467 | MTG, SFG |
| SOX2 | 213721_at | 1.961 | 61.061 | MTG, SFG |
| ZNF853 | 232884_s_at | 1.913 | 60.172 | MTG, SFG |
| ZNF358 | 219379_x_at | 1.887 | 55.688 | HIP, EC, PC, MTG, SFG |
| RXRA | 202449_s_at | 1.79 | 54.37 | EC, MTG, SFG |
| TCF7L2 | 236094_at | 1.928 | 54.141 | MTG, SFG |
| ZFHX4 | 219779_at | 2.038 | 53.88 | MTG, SFG |
| HMG20B | 210719_s_at | 1.954 | 52.728 | MTG, SFG |
| RBCK1 | 207713_s_at | 1.882 | 51.35 | HIP, SFG |
| ZCCHC24 | 212419_at | 1.84 | 50.551 | EC, MTG, SFG |
| ZDHHC21 | 229240_at | -1.778 | 49.702 | HIP, PC, MTG |
| BAZ2A | 201353_s_at | 2.148 | 48.675 | SFG |
| ZFYVE20 | 1553570_x_at | 2.045 | 48.522 | HIP, EC, MTG, SFG |
| ZNF711 | 228988_at | -1.745 | 48.184 | EC, MTG, SFG |
| ZNF529 | 231940_at | -1.75 | 48.062 | EC, MTG |
| RELA | 201783_s_at | 1.899 | 46.497 | MTG, SFG |
| ZBED3 | 228402_at | 2.011 | 46.421 | EC, MTG, SFG |
| ZFYVE20 | 1553569_at | 1.976 | 45.605 | EC, SFG |
| BCL6 | 228758_at | 1.973 | 42.783 | MTG, SFG |
| NR2F6 | 209262_s_at | 2.102 | 42.759 | HIP, MTG |
| NFIA | 224970_at | 1.828 | 40.975 | HIP, EC, MTG, SFG |
| HIF3A | 219319_at | 1.789 | 40.506 | HIP, EC, MTG, SFG |
| RBPJ | 211974_x_at | 2.01 | 40.502 | EC, MTG, SFG |
| MAZ | 212064_x_at | 1.967 | 38.763 | HIP, EC, PC, SFG |
| ZNF684 | 244398_x_at | -1.932 | 38.34 | MTG |
| ZNF623 | 206188_at | -1.757 | 37.829 | EC, MTG |
| ZFP36L1 | 211962_s_at | 1.821 | 37.694 | EC, MTG, SFG |
| NFATC1 | 211105_s_at | 1.791 | 35.589 | MTG, SFG |
| HDGF | 216484_x_at | 1.945 | 35.089 | HIP, EC, SFG |

| | | | | |
|---|---|---|---|---|
| ZC3HAV1 | 213051_at | 1.858 | 35.085 | MTG, SFG |
| MTF1 | 205322_s_at | 2.014 | 34.847 | MTG, SFG |
| MITF | 207233_s_at | 2.109 | 34.494 | EC, MTG, SFG |
| ZNF382 | 1557260_a_at | -1.906 | 34.339 | EC, MTG, SFG |
| ZHX2 | 203556_at | 1.82 | 33.717 | EC, SFG |
| TCF7L1 | 221016_s_at | 1.914 | 33.698 | HIP, EC, MTG, SFG |
| LMO7 | 202674_s_at | -1.768 | 32.936 | EC, MTG, SFG |
| CBL | 225234_at | 2.008 | 32.452 | HIP, MTG, SFG |
| ILF3 | 217804_s_at | 2.001 | 32.357 | HIP |
| SOX8 | 226913_s_at | 2.004 | 32.263 | MTG, SFG |
| BLZF1 | 203840_at | -1.767 | 30.922 | EC, MTG |
| TFE3 | 212457_at | 2.137 | 30.9 | HIP, MTG, SFG |
| PHF14 | 204525_at | -1.762 | 30.766 | MTG |
| LHX2 | 206140_at | 2.013 | 30.317 | MTG, SFG |
| STAT3 | 208992_s_at | 2.022 | 29.256 | EC, MTG, SFG |
| BCL11A | 219497_s_at | -1.879 | 29.094 | EC |
| MTF2 | 209705_at | -1.767 | 28.201 | MTG |
| MEF2A | 214684_at | -1.935 | 27.941 | MTG |
| NFIC | 206929_s_at | 2.026 | 26.069 | HIP, EC, MTG, SFG |
| ZBTB20 | 205383_s_at | 1.872 | 24.561 | EC, MTG, SFG |
| ZMYM5 | 235620_x_at | -1.828 | 24.188 | MTG, SFG |
| ZCCHC7 | 226496_at | -1.779 | 23.229 | EC, MTG, SFG |
| ZNF320 | 229614_at | -2.039 | 23.225 | HIP, MTG |
| ZNF688 | 213527_s_at | 2.1 | 22.886 | SFG |
| BRPF1 | 204481_at | 2.029 | 22.843 | MTG, SFG |
| ZNF382 | 1561687_a_at | -1.971 | 22.757 | PC, SFG |
| ZC3H7B | 205877_s_at | 1.874 | 18.879 | SFG |
| NFIC | 213298_at | 2.206 | 14.986 | HIP, EC, MTG, SFG |
| NFIB | 209289_at | 2.186 | 8.949 | SFG |
| | | | | |
| Control versus NDAD | | | | |
| ZMYM6 | 213698_at | -1.981 | 57.013 | HIP, PC |
| RBPJ | 211974_x_at | -2.076 | 40.524 | HIP, EC, PC, MTG |
| ZFHX4 | 219779_at | -2.016 | 28.419 | EC, PC |
| ZBTB16 | 205883_at | -2.053 | 27.904 | HIP |
| AATF | 209165_at | -1.933 | 27.584 | HIP |
| NCOA1 | 210249_s_at | -2.082 | 25.328 | HIP |
| ATF4 | 200779_at | -2.029 | 24.265 | HIP |
| MEF2D | 225641_at | 2.067 | 20.019 | HIP, EC, PC, MTG |
| TCF4 | 222146_s_at | -2.084 | 19.465 | HIP |
| ZMYND11 | 202136_at | -2.06 | 18.42 | PC |
| ZCCHC2 | 219062_s_at | 2.043 | 11.007 | HIP, PC, MTG |
| ZMYM2 | 202778_s_at | 2.079 | 10.707 | EC |

MR master regulator, NES normalized enrichment score, VCX visual cortex, AD Alzheimer’s disease, NDAD non-demented Alzheimer’s disease
