# Supplementary material for: Assembly and Interrogation of Alzheimer’s Disease Genetic Networks Reveal Novel Regulators of Progression
Source: PLoS One. 2015 Mar 17;10(3):e0120352. doi: 10.1371/journal.pone.0120352 (PMC4363671; doi:10.1371/journal.pone.0120352)
Supplement: S8 Table — (PDF) [file pone.0120352.s014.pdf]

	Control	moderate AD	severe AD
Age of death (yr)	82.6 ± 1.8	86.4 ± 2.2	82.4 ± 2.5
Clinical diagnostic	No	Yes	Yes
CERAD<br>(neuritic plaque density)	A<br>(Sparse)	B<br>(Moderate)	C<br>(Frequent)
Braak stage	III-IV	V-VI	VI
NFT frequency<br>(frontal/parietal cortex)	None	Low<br>(3-6 NT/100x)	High<br>(> 7 NT/100x)

CERAD consortium to establish a registry for Alzheimer's disease,
NFT neurofibrillary tangles
